# Phosphorylation of Bok at Ser-8 blocks its ability to suppress IP_3_R-mediated calcium mobilization

**DOI:** 10.1186/s12964-024-02008-8

**Published:** 2025-01-14

**Authors:** Caden G. Bonzerato, Katherine R. Keller, Richard J. H. Wojcikiewicz

**Affiliations:** Department of Pharmacology, SUNY Upstate Medical University, Syracuse, NY 13210 USA

**Keywords:** Bok, Phosphorylation, IP_3_R, ER, GPCR, Calcium signaling, ER calcium leak, PKA, Bcl-2 family proteins

## Abstract

**Background:**

Bok is a poorly characterized Bcl-2 protein family member with roles yet to be clearly defined. It is clear, however, that Bok binds strongly to inositol 1,4,5-trisphosphate (IP_3_) receptors (IP_3_Rs), which govern the mobilization of Ca^2+^ from the endoplasmic reticulum, a signaling pathway required for many cellular processes. Also known is that Bok has a highly conserved phosphorylation site for cAMP-dependent protein kinase at serine-8 (Ser-8). Whether Bok, or phosphorylated Bok, has any direct impact on the Ca^2+^ mobilizing function of IP_3_Rs remains to be established.

**Methods:**

Bok Ser-8 phosphorylation was characterized using purified proteins, G-protein coupled receptor agonists that increase cAMP levels in intact cells, mass spectrometry, and immunoreactivity changes. Also, using mammalian cells that exclusively or predominately express IP_3_R1, to which Bok binds strongly, and a fluorescent Ca^2+^-sensitive dye or a genetically-encoded Ca^2+^ sensor, we explored how endogenous and exogenous Bok controls the Ca^2+^ mobilizing function of IP_3_R1, and whether Bok phosphorylation at Ser-8, or replacement of Ser-8 with a phosphomimetic amino acid, is regulatory.

**Results:**

Our results confirm that Ser-8 of Bok is phosphorylated by cAMP-dependent protein kinase, and remarkably that phosphorylation can be detected with Bok specific antibodies. Also, we find that Bok has suppressive effects on IP_3_R-mediated Ca^2+^ mobilization in a variety of cell types. Specifically, Bok accelerated the post-maximal decline in G-protein coupled receptor-induced cytosolic Ca^2+^ concentration, via a mechanism that involves suppression of IP_3_R-dependent Ca^2+^ release from the endoplasmic reticulum. These effects were dependent on the Bok-IP_3_R interaction, as they are only seen with IP_3_Rs that can bind Bok (e.g., IP_3_R1). Surprisingly, Bok phosphorylation at Ser-8 weakened the interaction between Bok and IP_3_R1 and reversed the ability of Bok to suppress IP_3_R1-mediated Ca^2+^ mobilization.

**Conclusions:**

For the first time, Bok was shown to directly suppress IP_3_R1 activity, which was reversed by Ser-8 phosphorylation. We hypothesize that this suppression of IP_3_R1 activity is due to Bok regulation of the conformational changes in IP_3_R1 that mediate channel opening. This study provides new insights on the role of Bok, its interaction with IP_3_Rs, and the impact it has on IP_3_R-mediated Ca^2+^ mobilization.

**Supplementary Information:**

The online version contains supplementary material available at 10.1186/s12964-024-02008-8.

## Introduction

The Bcl-2 (B-cell lymphoma 2) protein family is known to govern the intrinsic (mitochondrial) apoptosis pathway, which controls cell survival and death [1], but also plays significant non-apoptotic roles [2]. Bcl-2-related ovarian killer, Bok, is often grouped together with the pro-apoptotic proteins, Bak and Bax, due to its ability to induce mitochondrial outer membrane permeabilization (MOMP) when over-expressed and to permeabilize liposomes or artificial MOMs in cell-free systems [3–5]. However, studies demonstrating “killer” properties of *endogenous* Bok are very limited, since Bok knock-out (BKO) has minimal effects on apoptotic signaling [6–10]. Additionally, Bok is constitutively bound to inositol 1,4,5-trisphosphate receptors (IP_3_Rs) at the endoplasmic reticulum (ER) membrane [8, 11, 12], which will restrict its ability to participate in MOMP [3, 4]. Non-apoptotic roles of Bok have recently been identified, including regulation of Ca^2+^ homeostasis [13, 14], ER-mitochondria contact sites [14, 15], and mitochondrial dynamics [7, 16].

There are three IP_3_R isoforms, IP_3_R1, IP_3_R2, and IP_3_R3, which can assemble to form homo- and/or hetero-tetrameric channels that govern the release of Ca^2+^ from the ER lumen into the cytosol [17–19]. Bok binds to an unstructured and surface-exposed loop in IP_3_R1, which is also found in IP_3_R2, but not IP_3_R3, correlating with the inability of Bok to bind to IP_3_R3 [4, 11, 20]. Remarkably, Bok expression is highly dependent on IP_3_R1 and IP_3_R2, since without them, Bok is rapidly degraded by the ubiquitin proteasome pathway [8]. Other Bcl-2 family proteins (e.g., Bcl-2, Bcl-xL, Mcl-1, and Bcl2L10) have also been shown to interact with IP_3_Rs [21–28], but Bok by far has the highest binding affinity [20]. Despite the high affinity binding, there is currently little evidence that Bok can directly regulate IP_3_R Ca^2+^ channel activity [4].

The activity of individual Bcl-2 family members is primarily controlled by a range of protein-protein interactions, but also by post-translational modifications, particularly phosphorylation [29, 30]. For example, studies on Mcl-1, Bcl-2, and Bax have shown that phosphorylation can impact stability, protein-protein interactions, localization, and apoptotic roles [29–31]. Regarding Bok, proteomic profiling of rat kidney cells showed that Bok can be phosphorylated at serine-8 (Ser-8) by cAMP-dependent protein kinase (PKA) [32, 33]. Here, we confirm that Bok is phosphorylated at Ser-8 by PKA in vitro and in response to G protein-coupled receptor (GPCR) activation in a range of cell types in vivo, and explore the possible effects that Ser-8 phosphorylation has on Bok function, focusing on the regulation of IP_3_R1-mediated Ca^2+^ mobilization. We find that during GPCR-mediated IP_3_R1 activation, Bok accelerates the post-maximal decline in cytosolic Ca^2+^ concentration ([Ca^2+^]_C_) due to suppression of ER Ca^2+^ release and that this effect is reversed by Bok phosphorylation at Ser-8. This study reveals a new role for Bok and its phosphorylation in modulating IP_3_R activity.

## Materials and methods

### Materials

Mouse pituitary αT3 cells (WT, BKO, IP_3_R1 KO [7, 34]) were maintained at 37 °C under 5% CO_2_ in Dulbecco’s modified Eagle’s medium supplemented with 5% fetal bovine serum, 100 U/mL penicillin, and 100 µg/mL streptomycin. Human embryonic kidney cells, i.e., HEK IP_3_R1-3KO (HEK-3KO) [35] and HEK-IP_3_R1 [36], human HeLa cells (WT and BKO [16]), and human neuroblastoma SH-SY5Y cells [37] were cultured in the same medium, except with 10% serum. Rabbit antibodies used were: anti-IP_3_R1 and anti-IP_3_R2 [37], anti-IP_3_R1-3 [38], anti-Bok^A^ (Bok^A^), raised against amino acids 19–32 of mouse Bok and already well established [8, 9, 12], anti-Bok^B^ (Bok^B^), a newly created and purified [37] Bok antibody raised against amino acids 11–25 (AAEIMDAFDRSPTDK) of mouse Bok, anti-Bok^C^ (Bok^C^) raised against residues surrounding V88 of human Bok #86875, anti-Mcl-1 #D35A5, anti-RRXpS #9624, anti-Bcl-2 #50E3, anti-Bcl-xL #54H6, anti-caspase-3 #9662, and anti-phospho-CREB #87G3 (Cell Signaling Technology). Mouse monoclonal antibodies used were: anti-HA epitope clone HA11 (Covance), anti-IP_3_R3 #610313 (BD Transduction Labs), anti-p97 #10R-P104A (Fitzgerald). Horseradish peroxidase-conjugated secondary antibodies, protease inhibitors, Triton X-100, CHAPS, carbamylcholine (carbachol, CCh), gonadotropin-releasing hormone (GnRH), thapsigargin (Tg), pituitary adenylate cyclase-activating polypeptide (PACAP), ATP, and EGTA were from Sigma. CalyculinA (CalA) and Forskolin (Fsk) were from Enzo Life Sciences. Isoproterenol and prostaglandin E1 (PGE1) were from Cayman Chemical. Protein A-Sepharose CL-4B was from GE Healthcare. Linear, MW ~ 25,000 polyethylenimine (PEI) was from Polysciences Inc. Precision Plus™ Protein Standards, and SDS-PAGE reagents were from Bio-Rad. Lipofectamine 2000 and Opti-MEM were from ThermoFisher.

### Cell lysis, immunoprecipitation (IP), and immunoblotting

**Fig. 1:**
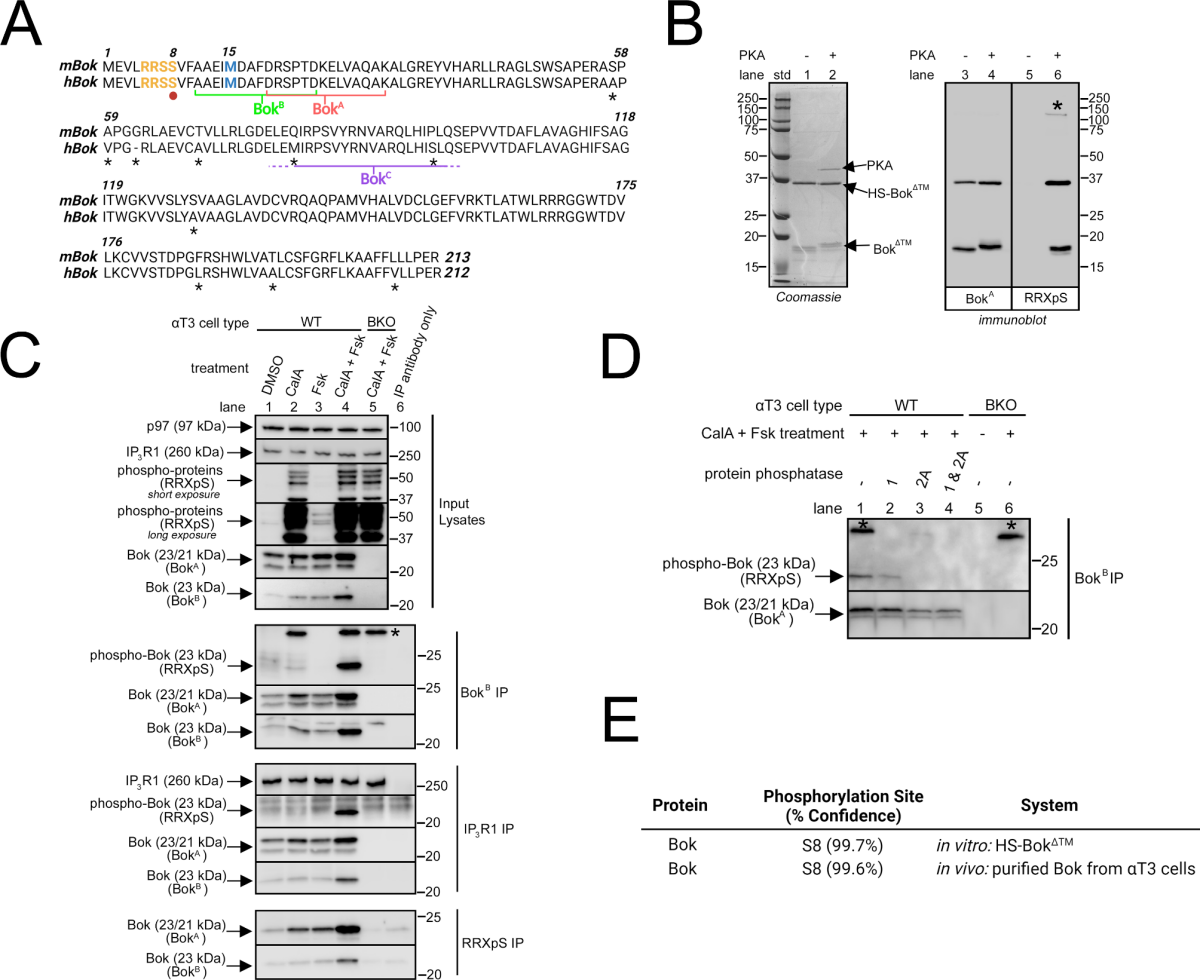
Bok is phosphorylated by PKA at Ser-8 in vitro and in vivo. (**A**) Amino acid sequences of mouse (m) and human (h) Bok (accession numbers O35425 and Q9UMX3, respectively) with amino acid differences labeled with asterisks. The PKA consensus sequence, RRSS, is highlighted in yellow and Ser-8 is labeled with a red dot. The epitopes for anti-Bok^A^ (Bok^A^) and anti-Bok^B^ (Bok^B^), as well as the approximate epitope for anti-Bok^C^ (Bok^C^) are indicated. In mammalian cells, Bok can be expressed as two forms: full-length Bok and varying levels of a shorter version, which results from alternative translation initiation at Met-15 (highlighted in blue). These forms migrate at 23/21 kDa in mouse cells and 22/20 kDa in human cells [8]. Bok^A^ recognizes both forms, while Bok^B^ preferably recognizes the full-length form. Bok^C^ only recognizes human Bok, most likely due to amino acid differences between mouse and human Bok in the Bok^C^ epitope region. (**B**) *Left panel*, Coomassie blue stain of purified, bacterially expressed HS-Bok^ΔTM^ incubated without or with the catalytic subunit of PKA (40 kDa). HS-Bok^ΔTM^ migrates at 36 kDa and a cleavage fragment (Bok^ΔTM^) migrates at 18 kDa. *Right panel*, immunoblot of the same samples probed with Bok^A^ and a PKA substrate antibody (RRXpS), with a contaminating phospho-protein at ~ 125 kDa in lane 6 labeled with an asterisk. (**C**) Immunoblots of lysates or immunoprecipitated Bok (using Bok^B^ or IP_3_R1) or PKA substrates (using RRXpS) from WT αT3 cells treated with 100 nM CalA and/or 20 µM Fsk for 10 min, probed for the proteins indicated (lanes 1–4). BKO αT3 cells treated with CalA and Fsk (lane 5) and each IP antibody only (lane 6) are negative controls, with a non-specific, phospho-protein at 30 kDa in the Bok^B^ IP labeled with an asterisk. (**D**) Immunoprecipitated Bok (using Bok^B^) from WT αT3 cells, treated as in Fig. 1C, lane 4, was incubated with 0.25 µM of PP1 and/or PP2A for 30 min at 37 °C. Samples were probed for the proteins indicated, with untreated (lane 5) and CalA and Fsk-treated (lane 6) BKO αT3 cells serving as negative controls. The 30 kDa phospho-protein, present in both WT and BKO cells (lanes 1 and 6, asterisk), is also dephosphorylated by the protein phosphatases (lanes 2–4) and serves as a control for the methodology. (**E**) Summary of MS data from HS-Bok^ΔTM^ phosphorylated by PKA in vitro and Bok purified via co-IP with IP_3_R1 from CalA and Fsk-treated WT αT3 cells

To prepare lysates for SDS-PAGE, cells were incubated at 4 °C for 30 min with ice-cold lysis buffer (1% Triton X-100, 150 mM NaCl, 50 mM Tris-HCl, 1 mM EDTA, 1 mM dithiothreitol, with the protease inhibitors 10 µM pepstatin A, 0.2 µM soybean trypsin inhibitor, 0.2 mM phenylmethylsulfonyl fluoride, and the phosphatase inhibitors 1 mM sodium orthovanadate, 10 mM sodium fluoride, 1 mM beta-glycerophosphate disodium, 1 mM sodium pyrophosphate, and 100 nM okadaic acid, pH 8.0) followed by centrifugation at 16,000 x g for 10 min at 4 °C. For IP and co-IP experiments, lysates were prepared the same way, except that 1% CHAPS was used instead of Triton X-100. Lysates were incubated with protein A-Sepharose CL-4B beads and antibodies for ~ 16 h at 4 °C, and IPs were washed three times with CHAPS lysis buffer. For dephosphorylation of endogenous Bok (Fig. 1D), Bok was immunopurified using Bok^B^ and eluted from beads by incubating with 0.1 mg/mL of Bok^B^ peptide at 4 °C for ~ 24 h. Eluted Bok was mixed 1:1 with phosphatase buffer (100 mM NaCl, 1 mM magnesium chloride, 50 mM HEPES, 1 mM dithiothreitol, pH 7.4) and with protein phosphatase 1 (PP1) (Novus Biologicals) and/or protein phosphatase 2 A (PP2A) (Cayman Chemical). For phosphorylation of purified, endogenous Bok (Fig. 8D), Bok-IP_3_R complex was immunopurified using anti-IP_3_R1, was resuspended in phosphorylation buffer (120 mM potassium chloride, 50 mM Tris Base, 1 mM magnesium chloride, 1 mM ATP, pH 7.2) and mixed with the catalytic subunit of PKA (Promega) (1:100 dilution) at 32 °C for 10 min. All cell lysate and IP samples were resuspended in gel loading buffer [39], incubated at 37 °C for 30 min, and subjected to SDS-PAGE and immunoblotting. Immunoreactivity was detected using Pico Chemiluminescent Substrates (ThermoFisher #34579) and a ChemiDoc imager (Bio-Rad).

### Mass spectrometry (MS) analysis

For in vitro Bok phosphorylation, bacterially expressed His-SUMO Bok^ΔTM^ (HS-Bok^ΔTM^) was purified as described [20]. 200 µL of HS-Bok^ΔTM^ (~ 20 µg) was diluted with 800 µL phosphorylation buffer and mixed with the catalytic subunit of PKA (1:100 dilution) at 32 °C for 10 min. The reaction was then stopped with 0.4% SDS and 10 mM dithiothreitol and the sample was stored at -20 °C. For in vivo Bok phosphorylation, ~ 1 × 10^9^ WT αT3 cells were treated with 100 nM CalA and 20 µM Fsk for 10 min, lysed with CHAPS lysis buffer as described above and Bok was immunopurified via co-IP with anti-IP_3_R1. The IP was then subjected to SDS-PAGE, stained with Coomassie blue G-250 (Sigma), and the region containing Bok (~ 20–25 kDa) was excised from the gel and stored at -20 °C. Both in vitro and in vivo samples were analyzed by the Upstate Proteomics Core, where an in-solution (in vitro) or in-gel [40] (in vivo) trypsin digestion of polypeptides (adapted from Promega #V5280 and Pierce #89871, respectively) [41] was performed as previously described [16]. The dried, trypsin-digested peptides were dissolved in 100 µL of solvent A (50% ACN, 0.1% TFA in water) and mixed with 15.6 mg of CaTiO_3_ to enrich phosphorylated peptides [42]. The mixture was diluted to 250 µL with solvent A, rotated end-over-end for 1 h at room temperature, washed three times with solvent A, and phosphopeptides bound to CaTiO_3_ were eluted twice with 200 µL of solvent B (10% NH_4_OH in water), each time allowing a 2 min incubation. The two eluates were combined, acidified immediately with 40 µL of 0.1% TFA and dried. The phosphopeptides were dissolved in 100 µL of 0.1% TFA, cleaned as described [16], dissolved in 20 µL of liquid chromatography loading solvent with 2 µL injected into an Orbitrap Fusion Lumos Liquid Chromatography-Mass Spectrometer (Thermo), and MS data was searched and analyzed as described [16].

### Generation and analysis of Bok and IP_3_R cDNAs

Wild-type (WT) human (h) and mouse (m) Bok cDNAs [12] were used to generate Bok Ser-8 mutants (Bok^S8A^ and Bok^S8E^) by site-directed mutagenic PCR. IP_3_R1HA^WT^, IP_3_R1HA^S1588A/S1755A^, IP_3_R1HA^Δ1916/17^, IP_3_R2, and IP_3_R3 cDNAs were generated as described [20, 43, 44]. Multiple independent cDNA preparations were used in experiments and their authenticity was confirmed by DNA sequencing (Genewiz). Primer sequences are available upon request. HEK-3KO cells [35] seeded at 6 × 10^5^/9.6 cm^2^ well were transiently transfected with Bok cDNAs (or an equivalent amount of pcDNA3 vector as control) together, in most experiments, with IP_3_R1HA cDNA, and 6 µl of 1 mg/mL PEI (pre-mixed in 50 µl of serum-free cultured medium). ~24 h later, cells were subcultured into Poly-D Lysine-Treated 96-well plates (Greiner) to measure [Ca^2+^]_C_ or to new 6-well plates for measurement of protein expression, or otherwise were harvested with ~ 0.2 mL/well lysis buffer and analyzed via immunoblotting or subjected to IP. Stably transfected cells were obtained by transfecting HEK-IP_3_R1 cells [36] with 0.25 µg Bok cDNAs or pcDNA3 vector and PEI as described above, or by transfecting BKO αT3 cells with Bok^WT^ or vector via electroporation using the Neon^®^ Transfection System (Invitrogen) as described [45]. ~24 h later, cells were selected in 3 mg/mL G418 for 72 h and then maintained in 1 mg/mL G418.

### [Ca^2+^]_C_ measurements

HEK-3KO and HEK-IP_3_R1 and cells were seeded into Poly-D Lysine-Treated 96-well plates at ~ 1 × 10^5^ cells/well and αT3 cells were seeded into regular 96-well plates (Corning) at ~ 1.3 × 10^5^ cells/well. To measure [Ca^2+^]_C,_ the FLIPR Calcium 6 (Cal6) assay kit (Molecular Devices) was used according to the manufacturer’s protocol, in which the cells were pre-incubated with Cal6 for 2 h. Cal6 fluorescence intensity (F) at 485 nm was detected using a FlexStation3 Multi-Mode Microplate Reader (Molecular Devices) and was normalized to the basal (initial) F value (F_0_) and graphed as F/F_0_, with the peak [Ca^2+^]_C_ response defined as the maximal F/F_0_ value (F_max_). To estimate the rate of post-maximal decline in [Ca^2+^]_C_, post-maximal values were graphed as F/F_max_ and the post-maximal area under the curve (AUC) expressed in arbitrary units (A.U.), and/or time to F/F_max_ = ~ 0.5 (the value chosen depended on the extent to which F/F_max_ declined, e.g., 0.6 for Figs. 4 and 5 and 0.5 for Fig. 6) were calculated.

### ER Ca^2+^ concentration ([Ca^2+^]_ER_) measurements

HEK-IP_3_R1 cells were seeded at 6 × 10^5^/9.6 cm^2^ well and transfected with R-CEPIAer cDNA (Addgene #58216) [46] and PEI. For αT3 cells, 3 × 10^6^ cells were transfected with R-CEPIAer cDNA via electroporation using the Neon^®^ Transfection System (Invitrogen) as described [45]. The following day, cells were seeded into Poly-D Lysine-Treated 96-well plates (~ 1 × 10^5^ HEK-IP_3_R1 cells/well) or regular 96-well plates (~ 1.3 × 10^5^ αT3 cells/well) and ~ 24 h later, culture media was changed to phenol-red free DMEM (Gibco). Fluorescence of R-CEPIAer in both HEK-IP_3_R1 and αT3 cells was initially detected using an EVOS Imaging System with an RFP light cube (ThermoFisher) at 10X objective to ensure equal transfection efficiency among cell lines. To measure [Ca^2+^]_ER_ in HEK-IP_3_R1 and αT3 cells, the R-CEPIAer fluorescence intensity (F) at 560 nm was detected using a FlexStation3 and normalized to initial F values (F_0_) and graphed as F/F_0_. For αT3 cells, the lowest F/F_0_ value after GnRH addition was defined as F_min_ and the rate of post-minimal recovery in [Ca^2+^]_ER_ was estimated by graphing post-minimal values as F/F_min_ and calculating the post-minimal AUC and time to F/F_min_ = 1.3 (~ 50% of the maximal recovery in F/F_min_). The rate of decline in [Ca^2+^]_ER_ after Tg addition was used to estimate ER Ca^2+^ leak by calculating time to F/F_0_ = 0.7 for HEK-IP_3_R1 cells and time to F/F_t=300_ = 0.6 for αT3 cells, both of which are ~ 50% of the maximal decline in [Ca^2+^]_ER_.

### Data presentation and analysis

All experiments were repeated at least twice and representative images of immunoblots with molecular markers (in kDa) on the side are shown. Immunoreactivity quantification was performed using ImageLab software (BioRad). Calcium traces shown are the mean fluorescent signals from multiple wells from an individual representative experiment. Quantitated data are expressed as mean ± SEM (n = the number of independent experiments) and calculations to estimate the rates of decline or recovery were done in GraphPad Prism. Statistical analysis was also performed in Prism using Student’s t-test (with Welch’s correction when variances are not assumed to be equal) or one-way ANOVA when 3 or more comparisons were performed (p-values of < 0.05 were considered statistically significant and denoted with “*”, while p-values > 0.05 were not considered statistically significant and denoted with “ns”). Figures 1A and 11 were created with Biorender.com.

## Results

### Bok is phosphorylated by PKA at Ser-8

PKA typically phosphorylates proteins at serine or threonine residues within the consensus sequence RRXS/T [47]. Ser-8 of Bok lies within a PKA consensus sequence highly conserved among various species, including mouse and human (Fig. 1A). To initially examine whether Bok is phosphorylated by PKA in vitro, purified, bacterially-expressed His-SUMO Bok^ΔTM^ (HS-Bok^ΔTM^) [20] was incubated with the catalytic subunit of PKA and probed with a PKA substrate antibody (RRXpS), which can detect proteins containing a phospho-serine with arginine in the − 3 and − 2 positions. The purified material migrates as two bands: intact HS-Bok^ΔTM^ at 36 kDa and a fragment lacking the HS tag (Bok^ΔTM^) at 18 kDa (Fig. 1B, lanes 1 and 3). Both proteins were phosphorylated when incubated with PKA (Fig. 1B, lane 6). Phosphorylation also caused a slight increase in the apparent molecular weight of Bok^ΔTM^ (Fig. 1B, lanes 2 vs. 1 and 4 vs. 3), and surprisingly, an increase in the immunoreactivity of HS-Bok^ΔTM^ and Bok^ΔTM^ when probed with Bok^A^ (Fig. 1B, lanes 4 vs. 3).

To examine whether Bok is also phosphorylated in vivo, αT3 cells, which contain relatively high levels of IP_3_R1 and Bok [8, 11], were exposed to the PP1/PP2A inhibitor, CalA, and/or the adenylate cyclase activator, Fsk, both of which increase the levels of generic phospho-proteins measured with RRXpS (Fig. 1C, input lysates panel, lanes 2–3) and the levels of phospho-CREB, a specific PKA substrate [48] (Supplemental Fig. 1C, lanes 2–3). CalA produced a much larger effect than Fsk, indicating that phosphatases are highly active, and the effects of CalA and Fsk were additive, confirming that Fsk is effective (both Figures, lanes 4). Two Bok specific antibodies, Bok^A^ and Bok^B^, both raised against the N-terminal of Bok near Ser-8 (Fig. 1A), were used to initially probe cell lysates and both showed increased immunoreactivity after exposure to CalA and/or Fsk, with the strongest effect seen when CalA and Fsk were used in combination (Fig. 1C, input lysates panel, lanes 2–4). These results are consistent with the immunoreactivity increase seen in Fig. 1B, lanes 4 vs. 3, and suggest that Bok phosphorylation increases the binding affinity of Bok^A^ and Bok^B^ for their epitopes. IP of Bok with Bok^B^, or via co-IP with IP_3_R1 [11], followed by probing with RRXpS, indicated that the strongest Bok phosphorylation was seen with CalA and Fsk in combination, and the same was true for the RRXpS IP of PKA substrates, followed by probing with Bok^A^ and Bok^B^ (Fig. 1C, lane 4). Both the in vivo data (Fig. 1C, lanes 2–4) and the in vitro data (Fig. 1B, lane 4 vs. 3) consistently show an increase in Bok^A^ and Bok^B^ immunoreactivity when Bok is phosphorylated. This was confirmed by exposing immunopurified, phosphorylated Bok to PP1 and PP2A. Both phosphatases reduced phospho-Bok recognition by RRXpS, showing that Bok is dephosphorylated, and produced a corresponding reduction in Bok^A^ immunoreactivity (Fig. 1D, lanes 2–4).

To show directly that Ser-8 of Bok is phosphorylated, both in vitro phosphorylated HS-Bok^ΔTM^ and in vivo phosphorylated Bok were analyzed by mass spectrometry (MS). This showed that Ser-8 was phosphorylated with > 99% confidence in both systems (Fig. 1E and Supplemental Files 1 and 2).

### Phosphorylation of endogenous Bok by GPCR activation

**Fig. 2:**
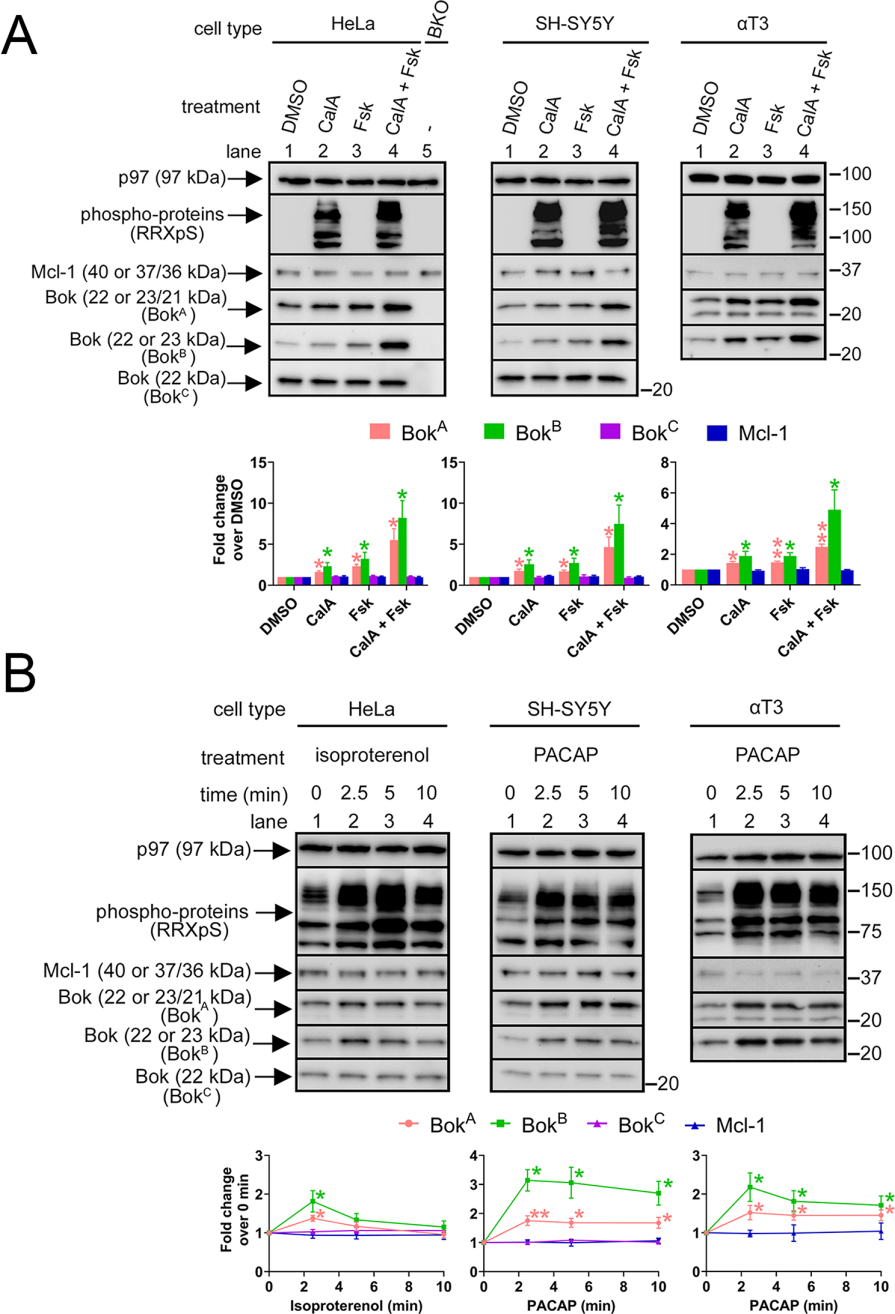
Phosphorylation of endogenous Bok, measured through changes in Bok immunoreactivity, is triggered by GPCR activation. (**A**) HeLa, SH-SY5Y, and αT3 cells were treated with DMSO, 100 nM CalA and/or 20 µM Fsk for 10 min and lysates were probed in immunoblots as indicated. HeLa BKO cells serve as a negative control for the Bok antibodies, probes for Mcl-1 and phospho-proteins serve as controls for the methodology, and p97 serves as a loading control. Histograms show Bok immunoreactivity seen with Bok^A^, Bok^B^, and Bok^C^, normalized to DMSO-treated cells (mean ± SEM, *n* = 5, * and ** designates *p* < 0.05 and *p* < 0.005 compared to DMSO-treated cells, respectively). (**B**) HeLa cells were treated with 10 µM isoproterenol, and SH-SY5Y and αT3 cells were treated with 100 nM PACAP for the times indicated. Lysates were probed in immunoblots as indicated, with Mcl-1 and phospho-proteins serving as controls for the methodology, and p97 serves as a loading control. Graphs show Bok immunoreactivity seen with Bok^A^, Bok^B^, and Bok^C^, normalized to 0 min levels (mean ± SEM, *n* = 5, * and ** designates *p* < 0.05 and *p* < 0.005 compared to 0 min levels, respectively)

**Fig. 3:**
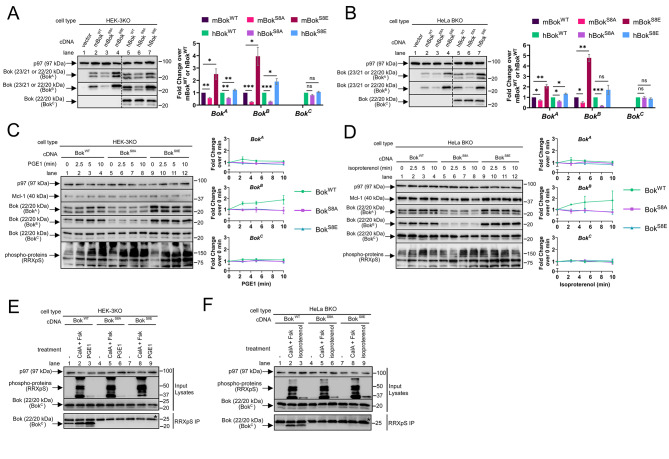
Bok mutants show that PKA-mediated Bok phosphorylation is limited to Ser-8. HEK-3KO and HeLa BKO cells were transfected to express mouse (m) or human (h) Bok^WT^, Bok^S8A^, or Bok^S8E^ (0.25 µg cDNAs). (**A** and **B**) Lysates were probed in the immunoblots as indicated, with p97 serving as a loading control. Histograms show immunoreactivity seen with Bok^A^, Bok^B^, and Bok^C^, normalized to mBok^WT^ or hBok^WT^ (mean ± SEM, *n* = 4, *, **, and *** designates *p* < 0.05, *p* < 0.005, and *p* < 0.0005, respectively, ns = not significant, *p* > 0.05). (**C** and **D**) HEK-3KO and HeLa BKO cells expressing hBok^WT^, hBok^S8A^, or hBok^S8E^ were treated with 1 µM PGE1 or 10 µM isoproterenol, respectively, for the times indicated. Lysates were probed in the immunoblots as indicated, with Mcl-1 and phospho-proteins serving as controls for the methodology, and p97 serves as a loading control. Histograms show Bok immunoreactivity seen with Bok^A^, Bok^B^ and Bok^C^, normalized to 0 min controls (mean ± SEM, *n* = 3). (**E** and **F**) Immunoblots of lysates or immunoprecipitated PKA substrates (RRXpS IP) from HEK-3KO and HeLa BKO cells expressing hBok^WT^, hBok^S8A^, or hBok^S8E^ exposed to 100 nM CalA and 20 µM Fsk for 10 min, or 1 µM PGE1 or 10 µM isoproterenol for 2.5 min. Samples were probed in the immunoblots as indicated, with p97 serving as a loading control and IgG light chain (25 kDa) is labeled with an asterisk

To further characterize endogenous Bok phosphorylation, we exploited the increase in Bok immunoreactivity seen with Bok^A^ and Bok^B^ when Bok is phosphorylated (Fig. 1C). We also employed Bok^C^, whose epitope is distant from Ser-8 (Fig. 1A), although this antibody recognizes human Bok, but not mouse Bok (see Fig. 3A and B), most likely due to amino acids differences in the antibody epitope (Fig. 1A). Treatment of human HeLa and SH-SY5Y cells, and αT3 cells with CalA and/or Fsk increased Bok immunoreactivity seen with Bok^A^ and Bok^B^ in all cell types, with the biggest effect usually seen with the drug combination (Fig. 2A, lane 4 vs. lanes 2 and 3), consistent with changes in the levels of generic phospho-proteins and phospho-CREB (Supplemental Fig. 1). In contrast, there was no change in Bok immunoreactivity seen with Bok^C^ in HeLa and SH-SY5Y cells, indicating that the binding affinity of Bok^C^ for Bok is unaffected by Bok phosphorylation, presumably because the Bok^C^ epitope is far from Ser-8. Likewise, the immunoreactivity of Mcl-1, which is well-known to be heavily phosphorylated [31] was unaffected by the drugs (Fig. 2A). HeLa BKO cells (lane 5) validate that all three Bok antibodies are specific to Bok. Overall, these data indicate that increases in immunoreactivity seen with Bok^A^ and Bok^B^ can be used to monitor Bok phosphorylation in both human and mouse cells, with Bok^C^ serving as a control unaffected by phosphorylation.

We next examined whether Bok phosphorylation can be triggered by endogenous signaling pathways, e.g., via GPCR activation. HeLa cells were treated with the β-adrenoreceptor agonist isoproterenol, and αT3 and SH-SY5Y cells were treated with PACAP, both of which have been shown to increase cAMP levels and therefore, activate PKA [49–51]. Indeed, both agents increased phospho-protein levels seen with RRXpS (Fig. 2B) and phospho-CREB levels, similar to that seen with Fsk (Supplemental Fig. 1). These agents also increased Bok immunoreactivity seen with Bok^A^ and Bok^B^ (maximally at 2.5 min) without changing Bok^C^ immunoreactivity, indicating that Bok is rapidly phosphorylated (Fig. 2B, lanes 2). Similar to results in Fig. 2A, Mcl-1 immunoreactivity was unaffected by GPCR activation. Overall, these results show that endogenous Bok is phosphorylated by GPCR activation in various cell types.

### Mutation of Ser-8 blocks Bok phosphorylation

To confirm that phosphorylation of Ser-8 is responsible for the increase in endogenous Bok immunoreactivity seen with Bok^A^ and Bok^B^ (Figs. 1 and 2), Ser-8 was mutated to alanine (creating Bok^S8A^), which cannot be phosphorylated, or to glutamic acid (creating Bok^S8E^), which mimics phospho-serine [52]. Mouse (m) and human (h) Bok^WT^, Bok^S8A^, and Bok^S8E^ were expressed in HEK-3KO cells, which lack all three IP_3_R isoforms [35] and express very little endogenous Bok (Supplemental Fig. 2), as well as in HeLa BKO cells (Fig. 3A and B, respectively). Surprisingly, when probed with Bok^A^ and Bok^B^, the Bok^S8E^ constructs were significantly more immunoreactive than the Bok^WT^ constructs, while the Bok^S8A^ constructs were significantly less immunoreactive than the Bok^WT^ constructs (lanes 2–7). In contrast, the immunoreactivity of hBok^S8A^ and hBok^S8E^ seen with Bok^C^ was not significantly different from hBok^WT^ (lanes 5–7). These data reveal that the amino acid present at position 8 of Bok strongly influences immunoreactivity seen with Bok^A^ and Bok^B^.

Next, we examined whether the GPCR-induced increases in endogenous Bok immunoreactivity seen with Bok^A^ and Bok^B^ (Fig. 2B) are solely due to phosphorylation of Bok at Ser-8 by exposing HEK-3KO and HeLa BKO cells expressing hBok^WT^, hBok^S8A^, and hBok^S8E^ to the adenylate cyclase activator PGE1 [43, 44, 53], or isoproterenol (Fig. 3C and D, respectively). The immunoreactivity of Bok^WT^ seen with Bok^A^ and Bok^B^ increased rapidly (near-maximal by 2.5 min) (lanes 1–4), while the immunoreactivity of Bok^S8A^ and Bok^S8E^ was unaffected (lanes 5–12), as was the immunoreactivity of all Bok constructs seen with Bok^C^ (lanes 1–12). Measurement of generic phospho-proteins and phospho-CREB showed that PGE1 and isoproterenol were effective (Fig. 3C and D and Supplemental Fig. 1A and D). Further, IP of phospho-proteins with RRXpS followed by probing with Bok^C^ showed that Bok^WT^, but not Bok^S8A^ and Bok^S8E^, was phosphorylated in response to CalA and Fsk treatment or GPCR activation (Fig. 3E and F, lanes 1–3 vs. 4–9).

Overall, the data in Fig. 3 demonstrate that Ser-8 is the only amino acid of Bok phosphorylated upon PKA activation. Further, the immunoreactivity increase seen with Bok^A^ and Bok^B^ when Ser-8 is phosphorylated in Bok^WT^, or when Ser-8 is mutated to glutamic acid (Fig. 3), or when Ser-8 is phosphorylated in endogenous Bok (Figs. 1 and 2), shows that the characteristics of the amino acid at position 8 greatly influences immunoreactivity. This increase is greatest with Bok^B^, presumably because Ser-8 is closer to the epitope of Bok^B^ than Bok^A^ (Fig. 1A). Since the immunoreactivity of Bok^C^ is unaffected by modulation of the amino acid at position 8, the increase in immunoreactivity seen with Bok^A^ and Bok^B^ is not due to an increase in Bok levels. Therefore, moving forward, we used Bok^B^ to monitor Bok phosphorylation at Ser-8, with Bok^C^ serving as a control to demonstrate that Bok levels are unchanged.

### Bok modulates exogenous IP_3_R1-mediated Ca^2+^ release

**Fig. 4:**
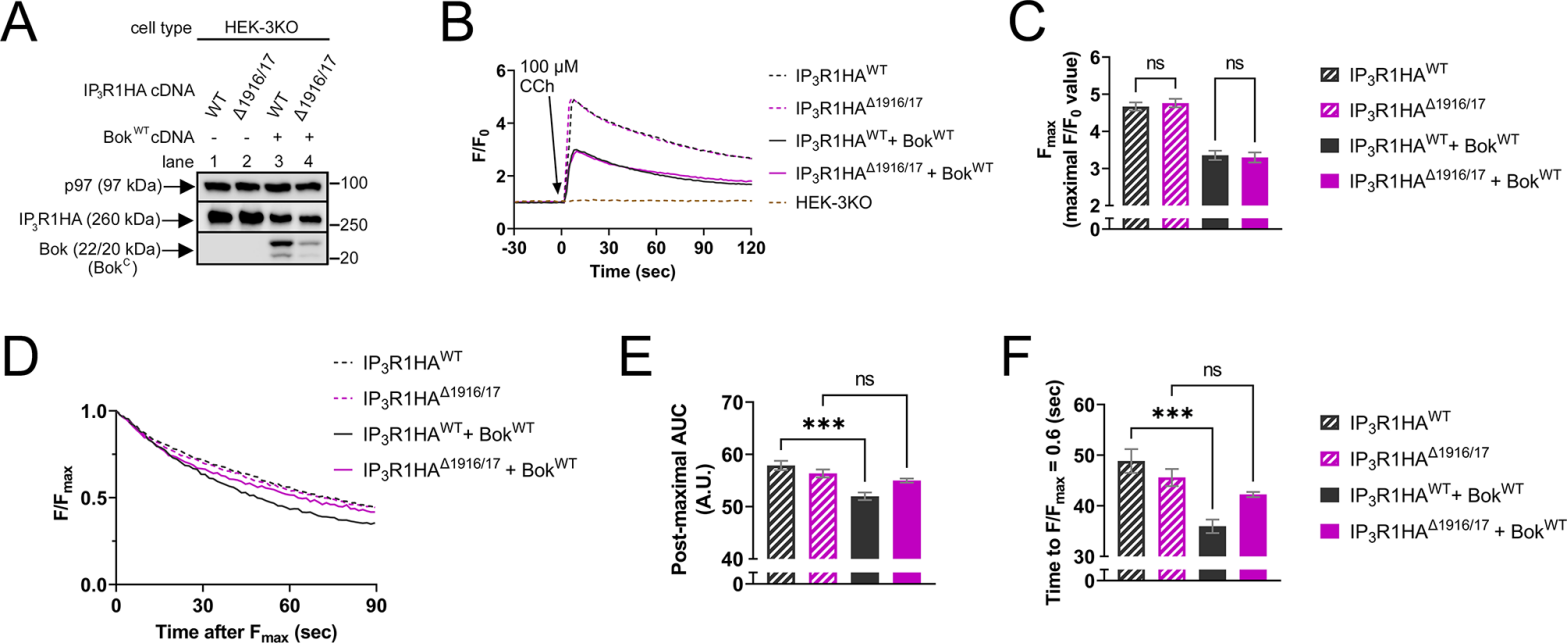
Exogenous Bok regulates exogenous IP_3_R1-mediated [Ca^2+^]_C_ responses in HEK-3KO cells. (**A**) HEK-3KO cells were transfected to express IP_3_R1HA^WT^ or IP_3_R1HA^Δ1916/17^ (2 µg cDNAs) with Bok^WT^ or vector (0.125 µg cDNA). Lysates were probed in immunoblots as indicated, with p97 serving as a loading control. (**B**) [Ca^2+^]_C_ (F/F_0_) in transfected HEK-3KO cells exposed to 100 µM CCh, added at t = 0. Parallel analysis of non-transfected HEK-3KO cells shows that [Ca^2+^]_C_ responses are due to exogenous IP_3_R1 expression. (**C**) Maximal F/F_0_ values (F_max_) (mean ± SEM, *n* = 3, ns = not significant, *p* > 0.05). (**D**) Post-maximal decline in [Ca^2+^]_C_ graphed as F/F_max_. (**E**) and (**F**) Post-maximal area under the curve (AUC) and time to F/F_max_ = 0.6 (mean ± SEM, *n* = 4, *** designates *p* < 0.0005, ns = not significant, *p* > 0.05)

Prior to exploring the functional effects of Bok phosphorylation, we examined whether Bok regulates IP_3_R1 function, using HEK-3KO cells transfected to express exogenous IP_3_R1 constructs with or without exogenous Bok^WT^ (Fig. 4A). The IP_3_R1 constructs were IP_3_R1HA^WT^ and the mutant IP_3_R1HA^Δ1916/17^, which due to deletion of amino acids 1916 and 1917, cannot bind Bok [20]. As expected, Bok^WT^ levels were higher when co-expressed with IP_3_R1HA^WT^ than IP_3_R1HA^Δ1916/17^ (lanes 3 vs. 4), because Bok stability is dependent on binding to IP_3_R1 [8, 20, 34]. Cells were exposed to the muscarinic receptor agonist CCh, which generates IP_3_ and triggers Ca^2+^ release from the ER [18, 36, 54]. Measurement of [Ca^2+^]_C_ (Fig. 4B and C) showed that there was no difference in the F_max_ between IP_3_R1HA^WT^ and IP_3_R1HA^Δ1916/17^ in the absence of Bok^WT^ (dotted lines and striped bars) or in the presence of Bok^WT^ (solid lines and bars). However, Bok^WT^ did cause a substantial reduction in F_max_ for both IP_3_R1 constructs (dotted lines and striped bars vs. solid lines and bars), but that correlated with a reduction in IP_3_R1 construct expression (Fig. 4A, lanes 3 and 4 vs. 1 and 2), most likely due to competition between IP_3_R1 and Bok^WT^ mRNAs for translational machinery when the cDNAs are co-expressed (described in more detail in Supplemental Fig. 3).

Interestingly, careful examination of the CCh responses revealed that when Bok^WT^ was present, the post-maximal decline in [Ca^2+^]_C_ was slightly more rapid in cells expressing IP_3_R1HA^WT^ than IP_3_R1HA^Δ1916/17^ (Fig. 4B, solid black vs. solid purple line). To quantify this difference, we graphed the post-maximal decline in F/F_max_ (Fig. 4D) and calculated the post-maximal AUC and time to F/F_max_ = 0.6 (Fig. 4E and F). IP_3_R1HA^WT^ co-expressed with Bok^WT^ had a significantly smaller post-maximal AUC and significantly shorter time to F/F_max_ = 0.6 when compared to IP_3_R1HA^WT^ alone (Fig. 4E and F, solid black vs. striped black bars). In contrast, these parameters were not significantly different for IP_3_R1HA^Δ1916/17^ in the presence or absence of Bok^WT^ (solid purple vs. striped purple bars). Overall, these data reveal that in CCh-stimulated HEK-3KO cells, exogenous Bok binding to exogenous IP_3_R1 accelerates the post-maximal decline in [Ca^2+^]_C_.

### Bok phosphorylation at Ser-8 modulates exogenous IP_3_R1-mediated Ca^2+^ release

**Fig. 5:**
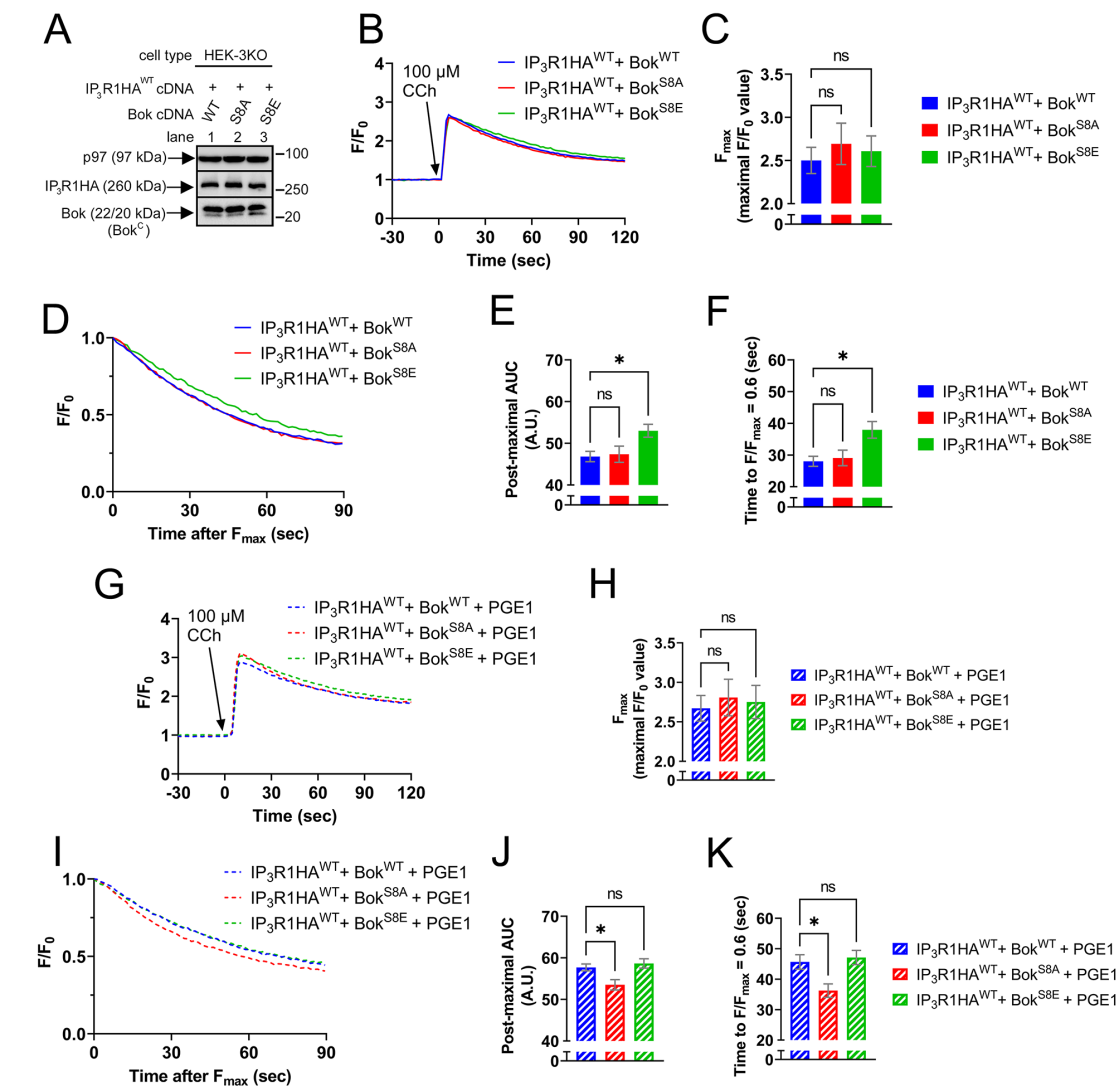
Bok phosphorylation at Ser-8 slows the post-maximal decline in exogenous IP_3_R1-mediated [Ca^2+^]_C_ responses in HEK-3KO cells. (**A**) HEK-3KO cells were transfected to express IP_3_R1HA^WT^ (2 µg cDNA) and either Bok^WT^, Bok^S8A^, or Bok^S8E^ (0.25 µg cDNAs). Lysates were probed in immunoblots as indicated, with p97 serving as a loading control. (**B**) [Ca^2+^]_C_ (F/F_0_) in transfected HEK-3KO cells exposed to 100 µM CCh, added at t = 0. Cal6 fluorescence intensity (F) was normalized to the basal (initial) F value (F_0_) and graphed as F/F_0_. (**C**) Maximal F/F_0_ values (F_max_) (mean ± SEM, *n* = 5, ns = not significant, *p* > 0.05). (**D**) Post-maximal decline in [Ca^2+^]_C_ graphed as F/F_max_. (**E**) and (**F**) Post-maximal area under the curve (AUC) and time to F/F_max_ = 0.6 (mean ± SEM, *n* = 5, * designates *p* < 0.05, ns = not significant, *p* > 0.05). (**G–K**) Parallel analysis of cells pretreated with 1 µM PGE1 for 2.5 min prior to CCh addition (mean ± SEM, *n* = 5, * designates *p* < 0.05, ns = not significant, *p* > 0.05)

Since Bok^WT^ accelerates the post-maximal decline in [Ca^2+^]_C_ (Fig. 4), we examined whether this effect is modified by Bok phosphorylation, initially using HEK-3KO cells transfected to express exogenous IP_3_R1HA^WT^ and either Bok^WT^, Bok^S8A^, or Bok^S8E^, with equal construct expression demonstrated with Bok^C^ (Fig. 5A). Exposure of cells to CCh and measurement of [Ca^2+^]_C_ (Fig. 5B) showed no significant differences in F_max_ values when comparing Bok^S8A^ or Bok^S8E^ to Bok^WT^ (Fig. 5C). Surprisingly, however, in comparison to Bok^WT^, the post-maximal decline in [Ca^2+^]_C_ was slower for Bok^S8E^ (Fig. 5B and D-F, blue vs. green lines and bars), whereas Bok^S8A^ was not significantly different from Bok^WT^ (red vs. blue lines and bars). Since Bok^S8E^ is phosphomimetic, these data suggest that phosphorylation at Ser-8 reverses the Bok^WT^-induced acceleration of the post-maximal decline in [Ca^2+^]_C_. These differential effects of the Bok constructs require IP_3_R binding, since IP_3_R2 was affected very similarly to IP_3_R1, while for constructs that cannot bind Bok (IP_3_R3 or IP_3_R1HA^Δ1916/17^) [11, 20], the post-maximal decline in [Ca^2+^]_C_ was identical in cells expressing Bok^WT^, Bok^S8A^, or Bok^S8E^ (Supplemental Fig. 4). Further, the differential effects cannot be explained by apoptotic signaling, since Bok over-expression in HEK-3KO cells caused only minimal increases in caspase-3 cleavage that were identical for the three constructs (Supplemental Fig. 5A).

To examine the effects of Bok phosphorylation at Ser-8 directly, cells were pre-treated with PGE1, which leads to the phosphorylation of Bok^WT^, but not Bok^S8A^ or Bok^S8E^ (Fig. 3C and E). Exposure of cells to CCh and measurement of [Ca^2+^]_C_ (Fig. 5G), again showed no significant differences in F_max_ values when comparing Bok^S8A^ or Bok^S8E^ to Bok^WT^ (Fig. 5H). Interestingly, after pre-treatment with PGE1, the post-maximal decline in [Ca^2+^]_C_ for Bok^WT^ became identical to Bok^S8E^ (Fig. 5I-K, striped blue vs. striped green lines and bars) and was now significantly slower than Bok^S8A^ (striped blue vs. striped red lines and bars). These results indicate that PGE1-induced phosphorylation of Bok^WT^ at Ser-8 slows the post-maximal decline in [Ca^2+^]_C_, similar to that seen with Bok^S8E^, and thus that phosphorylation blocks the ability of Bok to accelerate the post-maximal decline in [Ca^2+^]_C_.

Although not immediately obvious from the normalized data presented in Fig. 5, PGE1 had a general enhancing effect on CCh-induced increases in [Ca^2+^]_C_ (Supplemental Fig. 6A-E). This was seen with either Bok^WT^, Bok^S8A^, or Bok^S8E^, indicating that it was not due to Bok phosphorylation at Ser-8. Further, it was not due PKA-mediated IP_3_R1 phosphorylation at Ser-1588 and Ser-1755 [43], since IP_3_R1HA^S1588A/S1755A^ was affected by PGE1 just like IP_3_R1HA^WT^ (Supplemental Fig. 6F-K). Rather, an increase in ER Ca^2+^ store size appears to account for the enhancing effect of PGE1, since Tg, which releases the ER Ca^2+^ store [55], caused significantly larger increases in [Ca^2+^]_C_ in cells pre-treated with PGE1 than controls (Supplemental Fig. 6L-M).

### Endogenous IP_3_R1-mediated Ca^2+^ release is also modulated by Bok in a phosphorylation-dependent manner

**Fig. 6:**
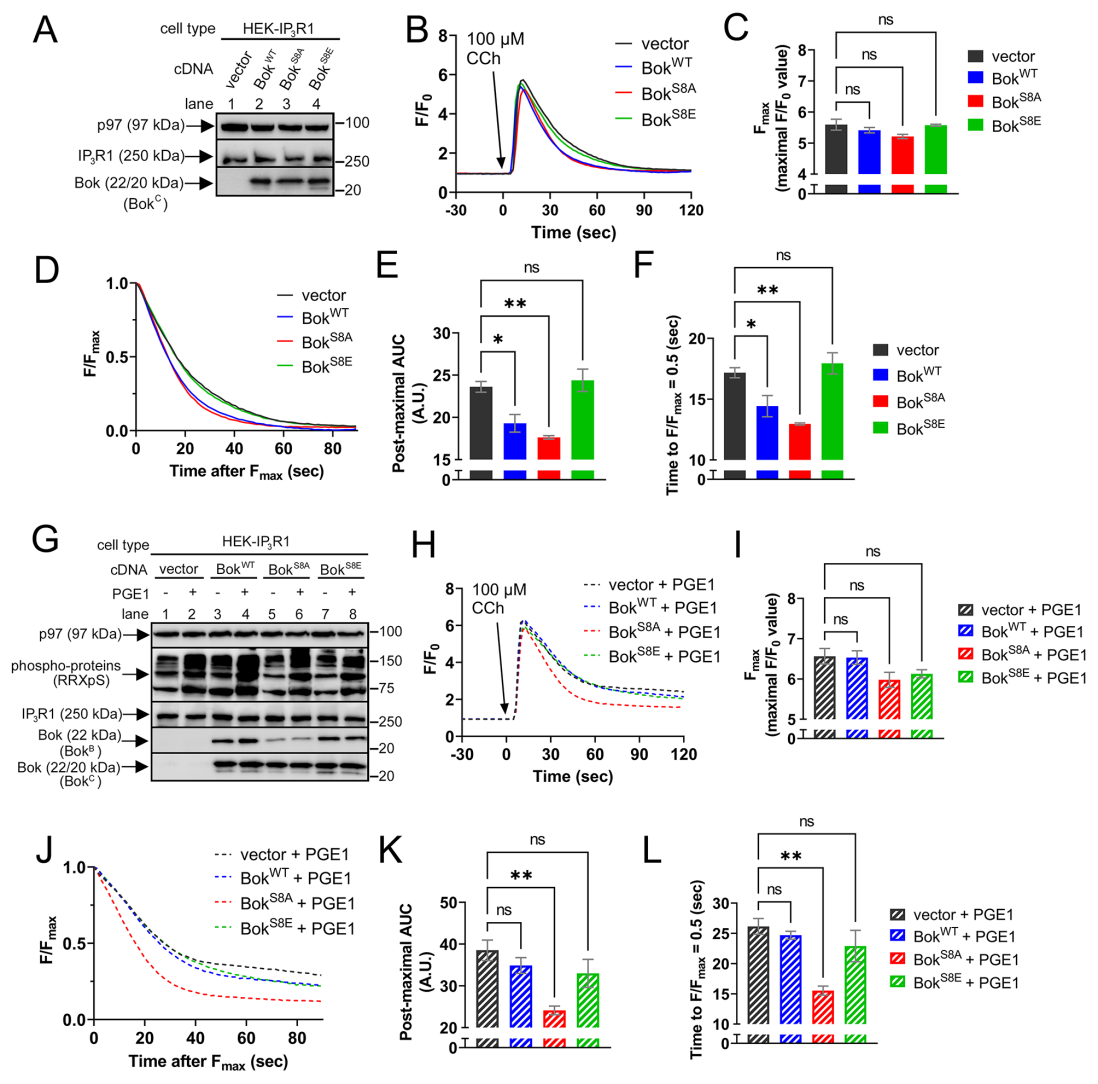
Exogenous Bok regulates endogenous IP_3_R1-mediated [Ca^2+^]_C_ responses in a Ser-8 phosphorylation-dependent manner in HEK-IP_3_R1 cells. (**A**) HEK-IP_3_R1 cells were transfected to stably express either Bok^WT^, Bok^S8A^, or Bok^S8E^, with vector as a control. Lysates were probed in immunoblots as indicated, with p97 serving as a loading control. (**B**) [Ca^2+^]_C_ (F/F_0_) in HEK-IP_3_R1 cells stably expressing the Bok constructs exposed to 100 µM CCh, added at t = 0. (**C**) Maximal F/F_0_ values (F_max_) (mean ± SEM, *n* = 3, ns = not significant, *p* > 0.05). (**D**) Post-maximal decline in [Ca^2+^]_C_ graphed as F/F_max_. (**E** and **F**) Post-maximal area under the curve (AUC) and time to F/F_max_ = 0.5 (mean ± SEM, *n* = 3, * and ** designates *p* < 0.05 and *p* < 0.005, respectively, ns = not significant, *p* > 0.05). (**G**) HEK-IP_3_R1 cells stably expressing Bok^WT^, Bok^S8A^, or Bok^S8E^, with vector as a control, were exposed to 1 µM PGE1 for 2.5 min and cell lysates were probed in immunoblots for the proteins indicated, with RRXpS serving as a control for the methodology and p97 as a loading control. (**H–L**) Parallel analysis of cells pretreated with 1 µM PGE1 for 2.5 min prior to CCh addition (mean ± SEM, *n* = 3, * and ** designates *p* < 0.05 and *p* < 0.005, respectively, ns = not significant, *p* > 0.05)

To extend these studies, we examined HEK-IP_3_R1 cells, which express endogenous IP_3_R1, but not IP_3_R2 and IP_3_R3 [36], and which like HEK-3KO cells, express very little endogenous Bok (Supplemental Fig. 2). Cells were transfected to stably express either Bok^WT^, Bok^S8A^, or Bok^S8E^, at approximately equal levels (Fig. 6A, lanes 2–4), with vector as a control (lane 1). Exposure of cells to CCh and measurement of [Ca^2+^]_C_ (Fig. 6B), showed no significant differences in F_max_ values when comparing either Bok^WT^, Bok^S8A^, or Bok^S8E^ to vector (Fig. 6C). However, in comparison to vector, the post-maximal decline in [Ca^2+^]_C_ was faster for Bok^WT^ and Bok^S8A^ (Fig. 6B and D-F, black vs. blue and red lines and bars), whereas Bok^S8E^ was not significantly different from vector (green vs. black lines and bars). Thus, again, Bok^WT^ accelerates the post-maximal decline in [Ca^2+^]_C_ (similar to Fig. 4) and this effect is not seen with Bok^S8E^ (similar to Fig. 5). Also again, apoptotic signaling cannot explain the differential effects of the stably expressed Bok constructs (Supplemental Fig. 5B).

Next, cells were pre-treated with PGE1, which leads to the phosphorylation of Bok^WT^ (Fig. 6G, lanes 3 vs. 4), but not Bok^S8A^ or Bok^S8E^ (lanes 5–8). Exposure of cells to CCh and measurement of [Ca^2+^]_C_ (Fig. 6H), again showed no significant differences in F_max_ values when comparing either Bok^WT^, Bok^S8A^, or Bok^S8E^ to vector (Fig. 6I). However, after pre-treatment with PGE1, the post-maximal decline in [Ca^2+^]_C_ (Fig. 6J-K) for Bok^WT^ was now identical to vector and similar to Bok^S8E^ (striped blue vs. striped black and green lines and bars), while Bok^S8A^ was still significantly faster compared to vector (striped red vs. striped black lines and bars). Overall, the data in Figs. 4, 5 and 6 show that in HEK cells expressing either exogenous or endogenous IP_3_R1, exogenous Bok accelerates the post-maximal decline in [Ca^2+^]_C_ and that phosphorylation of Bok at Ser-8 blocks this effect.

### Bok accelerates the post-maximal decline in [Ca^2+^]_C_ by suppressing IP_3_R1-mediated Ca^2+^ release from the ER

**Fig. 7:**
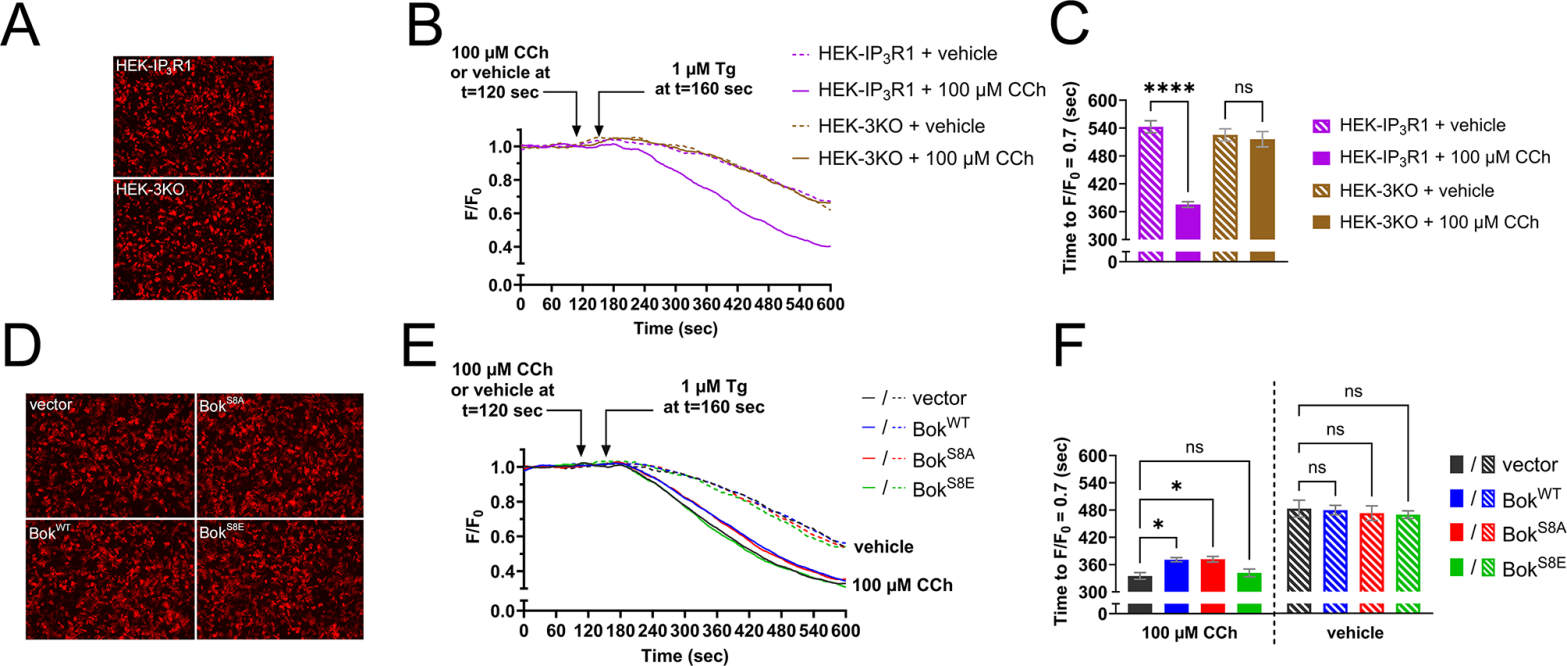
IP_3_R1-dependent ER Ca^2+^ leak is suppressed by Bok^WT^, but not Bok^S8E^. (**A**) HEK-IP_3_R1 and HEK-3KO cells were transfected to express R-CEPIAer with representative images of R-CEPIAer fluorescence shown. (**B**) [Ca^2+^]_ER_ (F/F_0_) in R-CEPIAer expressing cells exposed to 100 µM CCh or vehicle, added at t = 120 s, and 1 µM Tg, added at t = 160 s. (**C**) Rate of ER Ca^2+^ leak graphed as time to F/F_0_ = 0.7 (mean ± SEM, *n* = 3, **** designates *p* < 0.00005, ns = not significant, *p* > 0.05). (**D–F**) Parallel analysis of HEK-IP_3_R1 cells stably expressing Bok^WT^, Bok^S8A^, or Bok^S8E^, with vector as a control (mean ± SEM, *n* = 3, * designates *p* < 0.05, ns = not significant, *p* > 0.05)

To examine how Bok alters [Ca^2+^]_C_ in HEK-IP_3_R1 cells, we explored the possible involvement of the major modulators of [Ca^2+^]_C_ after GPCR activation, i.e., Ca^2+^ release from the ER, Ca^2+^ entry, and mitochondrial Ca^2+^ uptake [18, 56, 57]. Elimination of Ca^2+^ entry by pre-incubation with EGTA (extracellular Ca^2+^ chelator) [45], or inhibition of mitochondrial Ca^2+^ uptake by pre-incubation with MCU-i11 (inhibitor of the mitochondrial Ca^2+^ uniporter [57, 58]), did not affect the ability of Bok to accelerate the post-maximal decline in [Ca^2+^]_C_ (Supplemental Figs. 7 and 8, respectively), indicating that neither alteration of Ca^2+^ entry nor mitochondrial Ca^2+^ uptake is responsible for the acceleration. Next, the Ca^2+^ release properties of the ER were analyzed using the genetically-encoded ER Ca^2+^ sensor R-CEPIAer [46] (Fig. 7A). Also used was Tg, which by inhibiting SERCA activity, blocks replenishment of ER Ca^2+^ and allows for measurement of Ca^2+^ leak across the ER membrane [55]. Surprisingly, 100 µM CCh did not reduce [Ca^2+^]_ER_ in HEK-IP_3_R1 cells (Fig. 7B, purple solid line), indicating that only a very small amount of the total ER Ca^2+^ store accounts for the increase in [Ca^2+^]_C_ seen in Fig. 6. In contrast, Tg caused a gradual reduction in [Ca^2+^]_ER_ in HEK-IP_3_R1 and also in HEK-3KO cells (Fig. 7B and C, dashed lines and bars), and importantly, this reduction was accelerated by CCh in HEK-IP_3_R1 cells, but not HEK-3KO cells (solid vs. dashed lines and bars). Thus, while some Ca^2+^ leak across the ER membrane is IP_3_R1-independent, i.e., is the same in HEK-IP_3_R1 and HEK-3KO cells in the absence of CCh (dashed lines and bars), a considerable amount is IP_3_R1-dependent, since CCh accelerates Ca^2+^ leak in HEK-IP_3_R1 cells, where IP_3_R1 is present (purple solid vs. dashed lines and bars). Paradoxically, while the effects of CCh on [Ca^2+^]_C_ in HEK-IP_3_R1 cells were relatively short-lived (Fig. 6B), the acceleration of Ca^2+^ leak was long-lasting (Fig. 7B). This may be because Tg, by disrupting ER Ca^2+^ homeostasis and Ca^2+^-dependent feedback regulation of IP_3_Rs [17–19], traps activated IP_3_Rs at whatever their open state is when Tg is added. Support for this idea comes from studies showing that acute pre-treatment with Tg blocks IP_3_-induced conformational changes in IP_3_Rs that lead to recognition by the ubiquitin-proteasome pathway [59, 60].

To determine if this IP_3_R1-dependent ER Ca^2+^ leak is regulated by Bok and phosphorylation of Bok at Ser-8, HEK-IP_3_R1 cells stably expressing Bok^WT^, Bok^S8A^, and Bok^S8E^ were analyzed using R-CEPIAer, with vector as a control (Fig. 7D). Without CCh, ER Ca^2+^ leak was the same in all cell types (Fig. 7E and F, dashed lines and striped bars), indicating that in the absence of IP_3_R1 activation, ER Ca^2+^ leak is not dependent on Bok. As expected, IP_3_R1 activation with CCh accelerated Ca^2+^ leak in all cell types (dashed vs. solid lines), but interestingly Ca^2+^ leak was significantly slower in Bok^WT^ cells than vector cells (solid blue vs. black lines and bars), and slower ER Ca^2+^ leak was also seen with Bok^S8A^, but not with Bok^S8E^ (solid red and green lines and bars), suggesting that phosphorylation at Ser-8 reverses the suppressive effect of Bok on IP_3_R1-dependent Ca^2+^ leak. Overall, these data show that Bok suppresses IP_3_R1-dependent Ca^2+^ leak from the ER and this effect is reversed by Bok phosphorylation at Ser-8. In other words, Bok suppresses ER Ca^2+^ release through activated IP_3_R1 during CCh stimulation. This allows the ER to better retain Ca^2+^ that it absorbs from the cytosol after exposure to CCh, and likely explains why Bok accelerates the post-maximal decline in [Ca^2+^]_C_ seen in Fig. 6.

### Phosphorylation of Bok at Ser-8 weakens the Bok-IP_3_R1 interaction

**Fig. 8:**
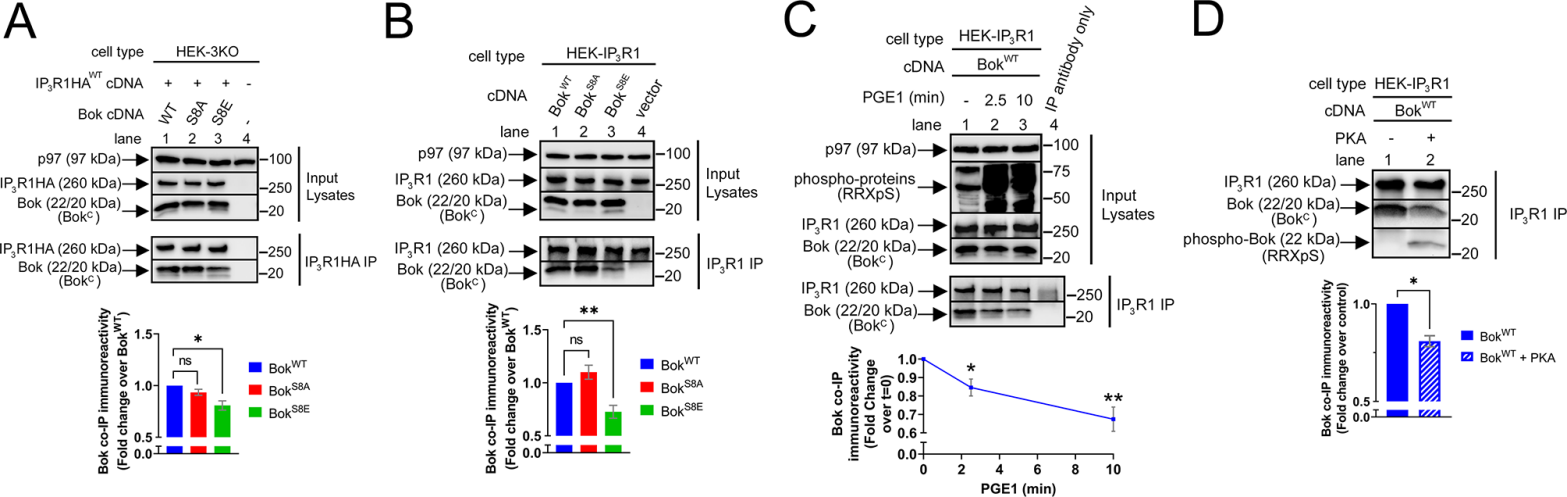
Bok^S8E^ or phosphorylation of Bok^WT^ at Ser-8 weakens the Bok-IP_3_R1 interaction. (**A**) HEK-3KO cells were transfected to express IP_3_R1HA^WT^ (2 µg cDNA) and either Bok^WT^, Bok^S8A^, Bok^S8E^, or vector (0.25 µg cDNAs). Immunoblots of lysates or IP_3_R1HA IPs were probed as indicated, with p97 serving as a loading control. Histogram shows co-IP immunoreactivity of the Bok constructs, normalized to Bok^WT^ (mean ± SEM, *n* = 4, * designates *p* < 0.05, ns = not significant, *p* > 0.05). (**B**) Immunoblots of lysates or IP_3_R1 IPs from HEK-IP_3_R1 cells stably expressing Bok^WT^, Bok^S8A^, or Bok^S8E^, with vector as a control, were probed as indicated, with p97 serving as a loading control. Histogram shows co-IP immunoreactivity of the Bok constructs, normalized to Bok^WT^ (mean ± SEM, *n* = 7, ** designates *p* < 0.005, ns = not significant, *p* > 0.05). (**C**) HEK-IP_3_R1 cells stably expressing Bok^WT^ were exposed to 1 µM PGE1 for 2.5 or 10 min. Immunoblots of lysates or IP_3_R1 IPs were probed as indicated, with RRXpS serving as a control for the methodology and p97 as a loading control. Histogram shows co-IP immunoreactivity of Bok, normalized to t = 0 (mean ± SEM, *n* = 8 for 2.5 min PGE1 and *n* = 5 for 10 min PGE1, * and ** designates *p* < 0.05 and *p* < 0.005, respectively). (**D**) IP_3_R1 IPs from HEK-IP_3_R1 cells stably expressing Bok^WT^ were incubated without or with the catalytic subunit of PKA. Immunoblots were probed as indicated, with RRXpS showing that Bok is phosphorylated. Histogram shows co-IP immunoreactivity of Bok, normalized to control (mean ± SEM, *n* = 3, * designates *p* < 0.05)

To examine why phosphorylation of Bok at Ser-8 reverses its effect on IP_3_R1 activity, we examined the interaction between exogenous IP_3_R1HA^WT^ and the Bok constructs via co-IP in HEK-3KO cells. Surprisingly, Bok^S8E^ bound significantly less well to IP_3_R1HA^WT^ than did Bok^WT^ (Fig. 8A, lanes 3 vs. 1, green vs. blue bars), while Bok^S8A^ bound equivalently to Bok^WT^ (lanes 2 vs. 1, red vs. blue bars). Similar results were seen in HEK-IP_3_R1 cells stably expressing the Bok constructs (Fig. 8B). Thus, introducing a phosphomimetic amino acid at position 8 of Bok weakens the Bok-IP_3_R interaction. Additional analysis of the IPs showed that neither Bok^WT^, nor the mutants, had any effect on potential interactions between Bcl-2 family proteins and the Bok-IP_3_R1 complex (Supplemental Fig. 9), ruling out such interactions as a reason for the differential effects of the Bok constructs on [Ca^2+^]_C_ and [Ca^2+^]_ER_ seen in Figs. 6 and 7.

To examine more directly whether phosphorylation of Bok at Ser-8 also weakens the Bok-IP_3_R1 interaction, HEK-IP_3_R1 cells stably expressing Bok^WT^ were incubated with PGE1, which phosphorylates Bok at Ser-8 (Fig. 6G). PGE1 significantly reduced Bok^WT^ co-IP with endogenous IP_3_R1 (Fig. 8C). Finally, incubation of immunopurified Bok-IP_3_R1 complex with PKA in vitro also inhibited the Bok-IP_3_R1 interaction (Fig. 8D). Overall, these data suggest that phosphorylation of Bok at Ser-8 or replacement of Ser-8 with a phosphomimetic amino acid weakens the Bok-IP_3_R1 interaction, which likely explains why Bok^S8E^ or phosphorylated Bok^WT^ are unable to accelerate the post maximal decline in [Ca^2+^]_C_ (Figs. 5 and 6) and why Bok^S8E^ is unable to suppress IP_3_R1-dependent ER Ca^2+^ leak (Fig. 7).

### Endogenous Bok also suppresses IP_3_R-mediated Ca^2+^ release from the ER

**Fig. 9:**
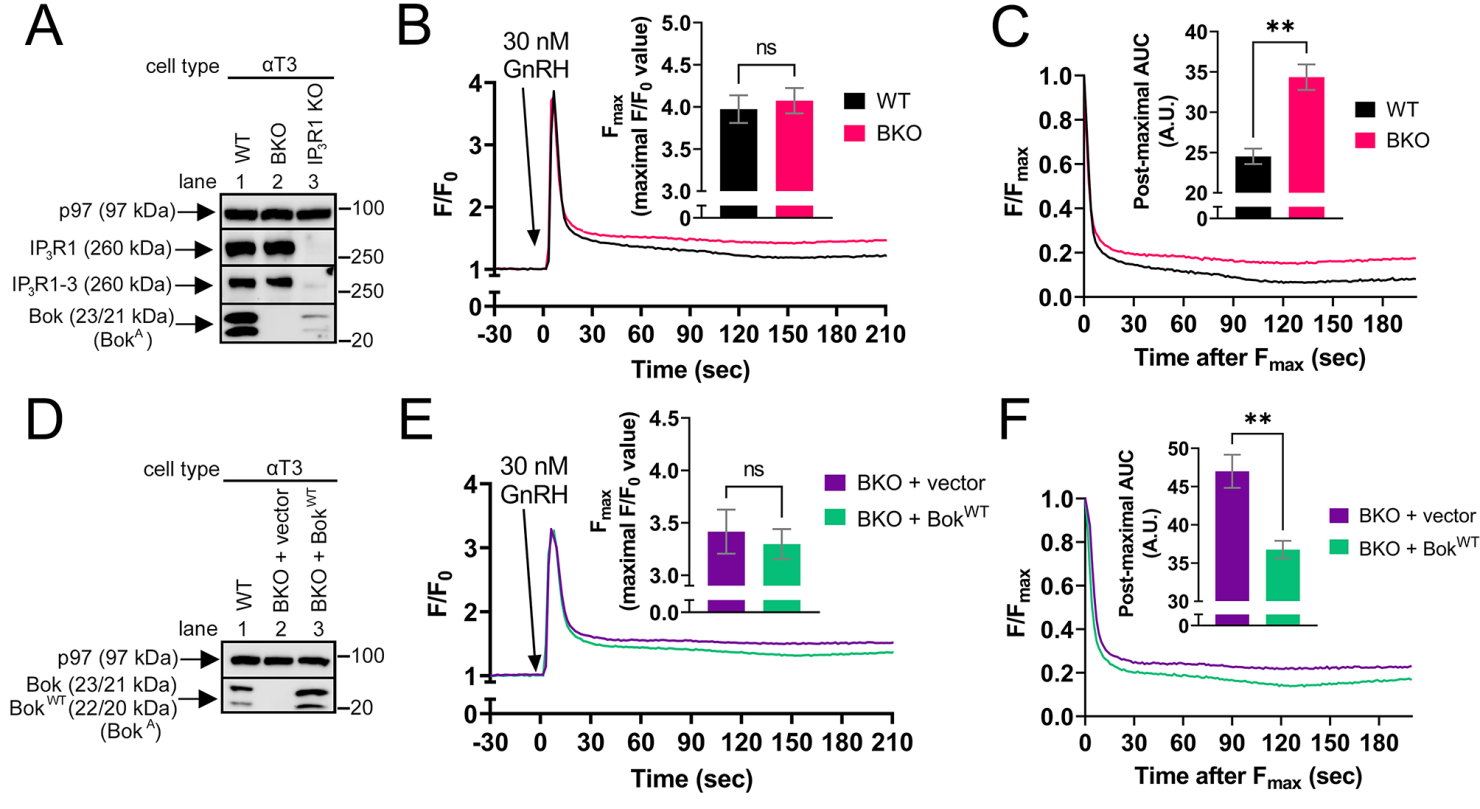
Endogenous Bok suppresses the post-maximal plateau phase of [Ca^2+^]_C_ in αT3 cells. (**A**) Lysates of WT, BKO, and IP_3_R1 KO αT3 cells were probed as indicated, with p97 serving as a loading control. Anti-IP_3_R1-3, which recognizes all three IP_3_R isoforms [38], demonstrates that IP_3_R1 is predominant in αT3 cells (lanes 1 vs. 3). (**B**) [Ca^2+^]_C_ (F/F_0_) in cells exposed to 30 nM GnRH, added at t = 0, with maximal F/F_0_ values (F_max_) graphed (mean ± SEM, *n* = 3, ns = not significant, *p* > 0.05). (**C**) Post-maximal decline in [Ca^2+^]_C_ graphed as F/F_max_ and post-maximal area under the curve (AUC) graphed (mean ± SEM, *n* = 3, ** designates *p* < 0.005). (**D**) WT αT3 cells and BKO αT3 cells stably transfected to express Bok^WT^, with vector as a control. Lysates were probed in immunoblots as indicated, with p97 serving as a loading control. (**E**) [Ca^2+^]_C_ (F/F_0_) in stably transfected cells exposed to 30 nM GnRH, added at t = 0, with maximal F/F_0_ values (F_max_) graphed (mean ± SEM, *n* = 5, ns = not significant, *p* > 0.05). (**F**) Post-maximal decline in [Ca^2+^]_C_ graphed as F/F_max_ and post-maximal area under the curve (AUC) graphed (mean ± SEM, *n* = 5, ** designates *p* < 0.005)

To examine whether endogenous Bok has effects similar to those seen in transfected HEK cells, we examined Ca^2+^ handling in WT and BKO cells that express predominately IP_3_R1, i.e., αT3 cells [34] (Fig. 9A) and SH-SY5Y cells [37, 61]. In these cells, depletion of IP_3_R1 dramatically reduces GPCR-induced [Ca^2+^]_C_ responses [60, 61], and any potential role of IP_3_R3, which is not regulated by Bok (Supplemental Fig. 4), can be ignored. Exposure of WT and BKO αT3 cells to GnRH generated a very rapid peak of [Ca^2+^]_C,_ with no significant differences in F_max_ values between WT and BKO cells, followed by a sustained “plateau” phase (Fig. 9B). While there was no discernible difference in the extremely rapid post-maximal decline of [Ca^2+^]_C_, the plateau phase was significantly elevated in BKO cells, as measured by an increase in the post-maximal AUC (Fig. 9C). This effect was reversed by stable expression of Bok^WT^ (Fig. 9D-F), indicating that the effect of Bok deletion is specific to Bok and not due to off-target effects. Also, endogenous Bok accelerated the post-maximal decline in [Ca^2+^]_C_ in SH-SY5Y cells (Supplemental Fig. 10). Thus, the data from αT3 cells and SH-SY5Y cells indicate that endogenous Bok has effects similar to those found with exogenous Bok in Figs. 4, 5 and 6.

**Fig. 10:**
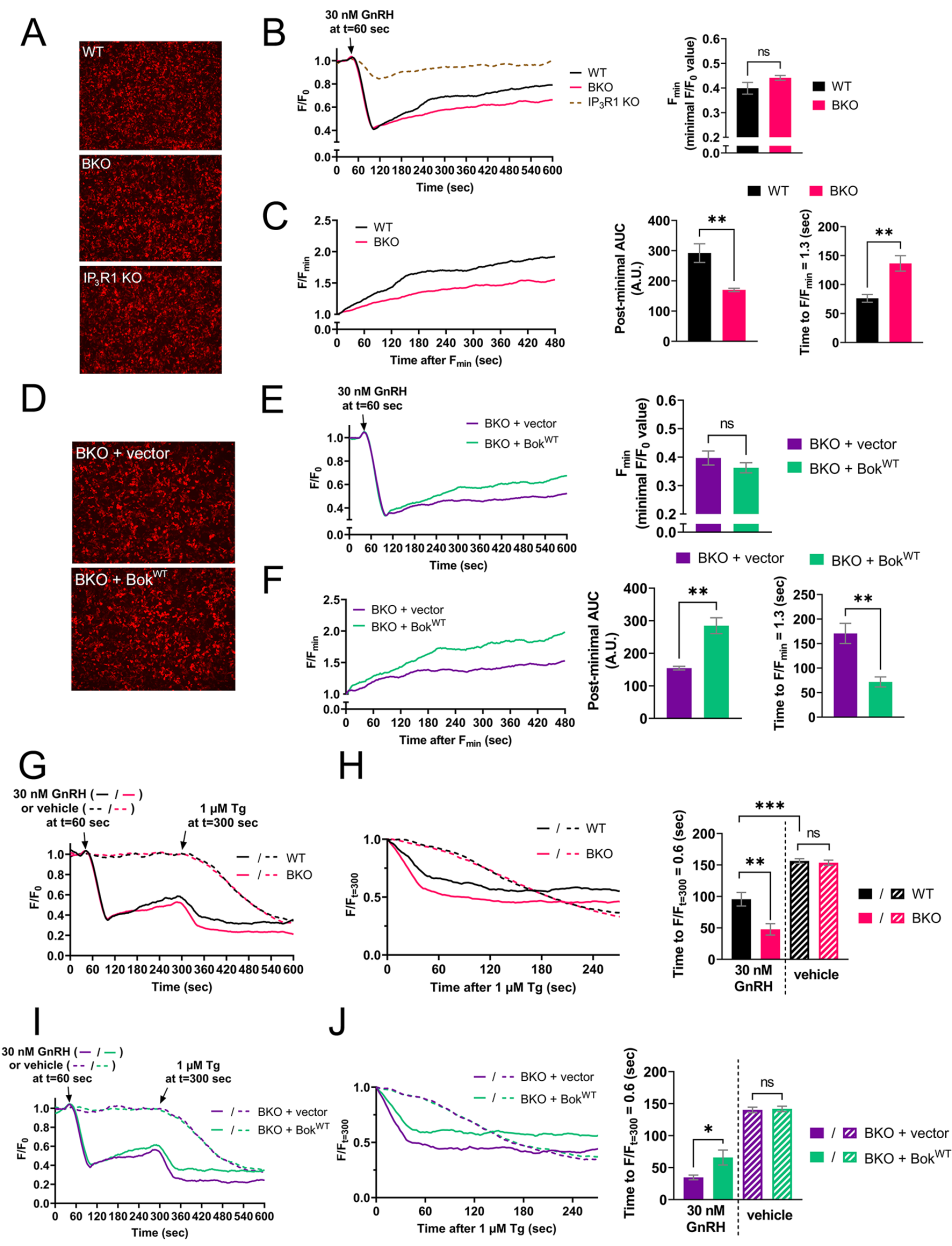
IP_3_R1-dependent ER Ca^2+^ leak is suppressed by endogenous Bok. (**A**) WT, BKO, and IP_3_R1 KO αT3 cells were transfected to express R-CEPIAer with representative images of R-CEPIAer fluorescence shown. (**B**) *Left*, [Ca^2+^]_ER_ (F/F_0_) in R-CEPIAer expressing cells exposed to 30 nM GnRH, added at t = 60 s. The lack of response to GnRH in IP_3_R1 KO cells (dotted line) shows that decreases in [Ca^2+^]_ER_ are due to endogenous IP_3_R1 expression. *Right*, minimal F/F_0_ values (F_min_) (mean ± SEM, *n* = 5, ns = not significant, *p* > 0.05). (**C**) *Left*, Post-minimal recovery in [Ca^2+^]_ER_ graphed as F/F_min_. *Right*, post-minimal area under the curve (AUC) and time to F/F_min_ = 1.3 (mean ± SEM, *n* = 5, ** designates *p* < 0.005). (**D–F**) Parallel analysis of BKO αT3 cells stably transfected to express Bok^WT^, with vector as a control (mean ± SEM, *n* = 4, ** designates *p* < 0.005, ns = not significant, *p* > 0.05). (**G**) [Ca^2+^]_ER_ (F/F_0_) in R-CEPIAer expressing cells exposed to 30 nM GnRH or vehicle, added at t = 60 s, and 1 µM Tg, added during the recovery phase of [Ca^2+^]_ER_ at t = 300 s (when [Ca^2+^]_ER_ partially recovered). (**H**) *Left*, decline in [Ca^2+^]_ER_ after 1 µM Tg addition normalized to F_t=300_ and graphed as F/F_t=300_. *Right*, rate of ER Ca^2+^ leak graphed as time to F/F_t=300_ = 0.6 (mean ± SEM, *n* = 6, ** and *** designates *p* < 0.005 and *P* < 0.0005, respectively, ns = not significant, *p* > 0.05). (**I–J**) Parallel analysis of BKO cells stably transfected to express Bok^WT^, with vector as a control (mean ± SEM, *n* = 5, * designates *p* < 0.05, ns = not significant, *p* > 0.05)

To examine how Bok alters the plateau phase of [Ca^2+^]_C_ in αT3 cells, the Ca^2+^ release properties of the ER were analyzed using R-CEPIAer (Fig. 10A). In contrast to HEK-IP_3_R1 cells (Fig. 7B), GPCR activation (with GnRH) substantially reduced [Ca^2+^]_ER_ (Fig. 10B) and this was dependent on IP_3_R1 since there was little change in [Ca^2+^]_ER_ in IP_3_R1 KO αT3 cells (dashed line). This reduction, or minimal response (F_min_), was the same in WT and BKO αT3 cells (right panel), but interestingly, [Ca^2+^]_ER_ recovered less well in BKO cells compared to WT (pink vs. black lines). To quantify this difference, we graphed the post-minimal recovery in F/F_min_ (Fig. 10C, left panel) and calculated the post-minimal AUC and time to F/F_min_ = 1.3 (right panels). BKO cells had a significantly smaller post-minimal AUC and a significantly longer time to time to F/F_min_ = 1.3 compared to WT (pink vs. black bars). The effect of Bok deletion on the recovery phase of [Ca^2+^]_ER_ was reversed by stable expression of Bok^WT^ in BKO αT3 cells (Fig. 10D-F), indicating that it is not due to off target effects. These data show that during IP_3_R1 activation, [Ca^2+^]_ER_ recovers less well in the absence of Bok, perhaps again because the ER is leakier.

To examine if endogenous Bok alters IP_3_R1-dependent Ca^2+^ leak from the ER in αT3 cells, cells were exposed to Tg during the recovery phase of [Ca^2+^]_ER_ (Fig. 10G). GnRH significantly increased Tg-induced Ca^2+^ leak from the ER (Fig. 10H, solid vs. striped black lines and bars), indicating that ER Ca^2+^ leak is much faster when IP_3_R1 is activated. This IP_3_R1-dependent Ca^2+^ leak was significantly faster in BKO cells compared to WT (solid pink vs. solid black lines and bars) and was reversed by stable expression of Bok^WT^ in BKO αT3 cells (Fig. 10I and J), indicating again that this effect is truly dependent on Bok. Overall, the data in Fig. 10 indicate that endogenous Bok suppresses ER Ca^2+^ release through activated IP_3_R1 during GnRH stimulation and this likely explains why BKO elevates the plateau phase (Fig. 9).

## Discussion

Our examination of the effects of Bok, and of Bok phosphorylation, on GPCR-induced Ca^2+^ signaling revealed that Bok accelerates the post-maximal decline in [Ca^2+^]_C_ during IP_3_R1 activation in cell systems expressing exogenous Bok (HEK-3KO and HEK-IP_3_R1 cells). This acceleration was due to Bok binding to IP_3_Rs, as it was not seen with receptors that do not bind Bok, and was reversed by PKA-induced phosphorylation of Bok at Ser-8 or phosphomimetic Bok^S8E^, rendering alternative explanations (e.g. regulation of IP_3_ metabolism, SERCA activity, etc.) highly unlikely. The effect on the post-maximal decline in [Ca^2+^]_C_ appears to be due to Bok suppression of Ca^2+^ release from the ER during IP_3_R1 activation. Similar results were observed for endogenous Bok in αT3 and SH-SY5Y cells. These results reveal new roles for Bok and Ser-8 Bok phosphorylation in controlling the Ca^2+^ mobilizing function of IP_3_Rs.

The main line of evidence that Bok regulation of ER Ca^2+^ release explains why Bok regulates the post-maximal phase of [Ca^2+^]_C_ is that endogenous and exogenous Bok suppress IP_3_R1-dependent ER Ca^2+^ leak, which was measured by treating cells with Tg shortly after GPCR stimulation. Tg blocks Ca^2+^ uptake into the ER [55] and thus, measuring [Ca^2+^]_ER_ after Tg addition provides an index of ER Ca^2+^ leak. Since Bok makes the ER less leaky during IP_3_R1 activation, the ER is better able to retain Ca^2+^ that it absorbs from the cytosol during the post-maximal phase. In HEK and SH-SY5Y cells, this causes an acceleration in the post-maximal decline in [Ca^2+^]_C_, whereas in αT3 cells it suppresses the post-maximal plateau phase. Another line of evidence is that the effects of Bok on both ER Ca^2+^ leak and the post-maximal phase of [Ca^2+^]_C_ were reversed when Bok was phosphorylated at Ser-8. Interestingly, Ser-8 phosphorylation reduced Bok co-IP with IP_3_R1, suggesting that disruption of Bok binding to IP_3_R1 (or the absence of Bok in BKO cells) enhances ER Ca^2+^ release through active IP_3_R1 channels.

**Fig. 11:**
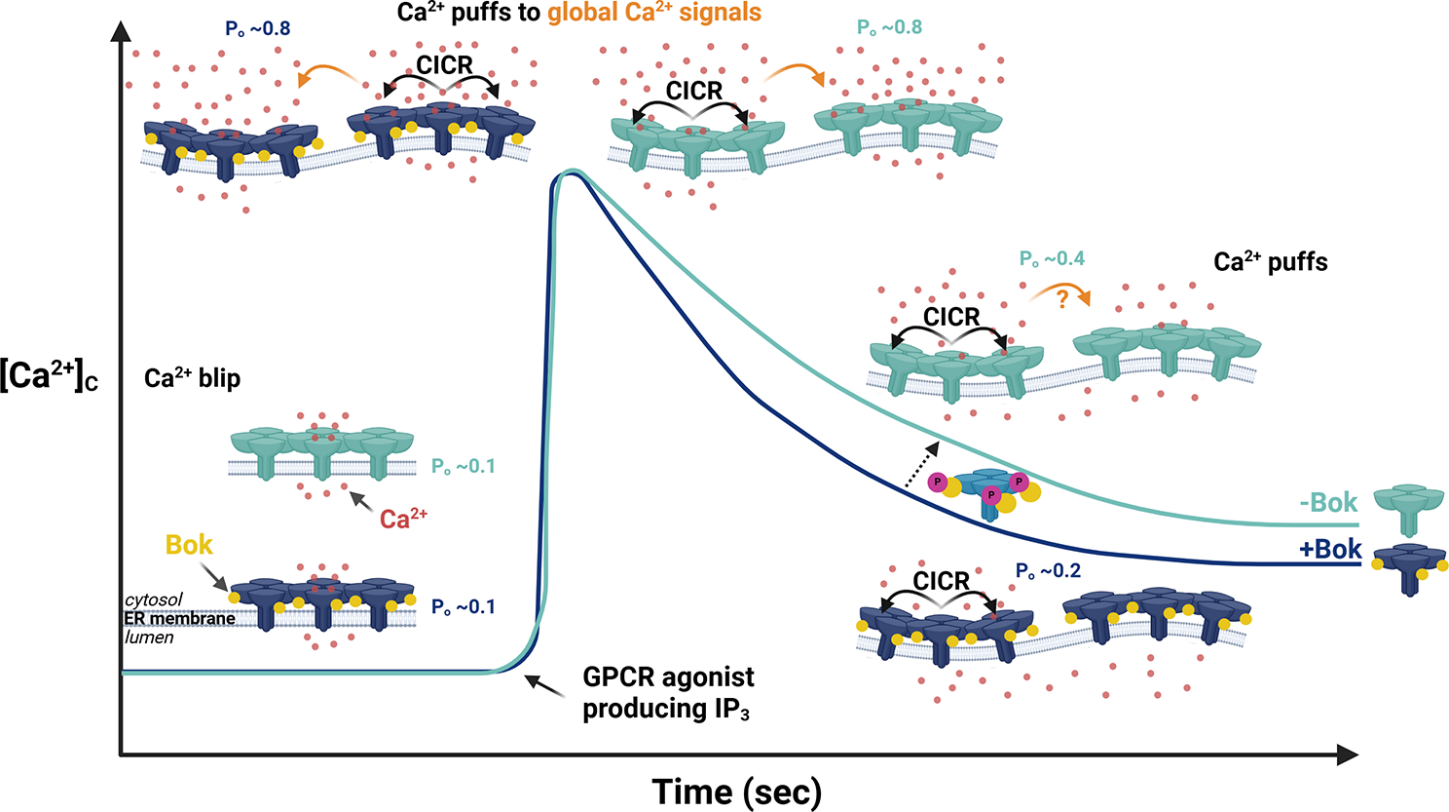
Model of Bok effects on IP_3_R1-mediated Ca^2+^ mobilization from the ER. GPCR-induced Ca^2+^ release from the ER can be explained in terms of clusters of IP_3_R1 leading to local Ca^2+^ blips (pre-GPCR stimulation), local Ca^2+^ puffs (moderate [IP_3_]), and global increases in [Ca^2+^]_C_ (high [IP_3_]), with each event reflecting a certain level of IP_3_R1 open probability (P_o_) [18, 62]. The [Ca^2+^]_C_ traces shown throughout this study and the model represent integrated Ca^2+^ release events from thousands of cells, either with Bok (dark blue) or without Bok (light blue). We speculate that Ca^2+^ blips dominate during the pre-stimulation phase (e.g., average P_o_ ~0.1), and that GPCR-induced maximal [Ca^2+^]_C_ is set by CICR-driven, global Ca^2+^ signals from numerous Ca^2+^ puffs throughout the cell (e.g., average P_o_ ~0.8). These Ca^2+^ release events are Bok-independent (i.e., are the same in the presence or absence of Bok). In contrast, the post-maximal phase of GPCR-induced Ca^2+^ mobilization, where P_o_ falls and [Ca^2+^]_C_ declines for a variety of possible reasons [18, 67, 68], is suppressed by Bok, presumably because Bok further decreases IP_3_R1 P_o_ (e.g., average P_o_ ~0.2 and ~ 0.4 in the presence or absence of Bok, respectively). This allows for a more rapid transition from global Ca^2+^ release events back to isolated Ca^2+^ puffs and/or blips, less Ca^2+^ release from the ER, and a more rapid decline in [Ca^2+^]_C_. Because Bok binds strongly to IP_3_R1, it is likely that it would regulate some aspect of IP_3_R1 activity [4]. Accordingly, Ser-8 phosphorylation of Bok (purple), which weakens its ability to interact with IP_3_R1, reverses the effect of Bok on IP_3_R activity and slows the post-maximal decline in [Ca^2+^]_C_

How does Bok regulate IP_3_R-mediated Ca^2+^ release from the ER? The answers to this question likely lie within the complex mechanisms that control IP_3_R open-probability (P_o_) and elementary and global IP_3_R-mediated Ca^2+^ release events [17–19, 62]. The elementary events are termed Ca^2+^ “blips” and “puffs” and result from the opening of a single IP_3_R tetramer (causing a blip), and at elevated [IP_3_], the coordinated opening of multiple IP_3_Rs tetramers within a cluster (causing a puff) [63–65]. Global IP_3_R-mediated Ca^2+^ increases occur at high [IP_3_] when numerous Ca^2+^ puffs activate IP_3_Rs throughout the cell [66]. After global [Ca^2+^]_C_ peaks, decreasing [IP_3_], phosphatidylinositol 4,5-bisphosphate depletion, and inhibitory effects of Ca^2+^ lower IP_3_R activity and consequently there is a transition back to isolated Ca^2+^ puffs and or/blips and a fall in [Ca^2+^]_C_ [18, 67, 68]. In the present study (modeled in Fig. 11), GPCR stimulation led to a rapid increase in [Ca^2+^]_C_ from basal to peak, that we presume represents a dramatic increase in IP_3_R1 P_o_ and a transition from Ca^2+^ blips (basal) to Ca^2+^ puffs and global Ca^2+^ release (peak) [67], and this was not dependent on Bok. In contrast, the post-maximal decline in IP_3_R1 P_o_ and transition back to isolated Ca^2+^ puffs and/or blips was enhanced by Bok, presumably because Bok further reduces IP_3_R1 P_o_ during this phase. Why Bok would only affect P_o_ during the post-maximal phase remains unclear. Perhaps, Bok cannot substantially oppose the multiple Ca^2+^-induced Ca^2+^ release (CICR) mechanisms that mediate the rapid, “firework”-like release events that produce global Ca^2+^ signals [36]. In contrast, during the post-maximal phase, when P_o_ is falling, IP_3_R1 activity is more moderate and susceptible to regulation [18]. During this phase, Bok could enhance Ca^2+^-dependent events that mediate IP_3_R1 inactivation or simply could inhibit the conformational changes that trigger IP_3_R1 channel opening [18, 19, 69]. That Bok may regulate IP_3_R1 P_o_ would not be unexpected, given that Bok binds with high affinity to IP_3_R1, most likely with four Bok proteins per IP_3_R1 tetramer [7, 20]. Measurement of IP_3_R1 P_o_ in control and BKO DT40 cells using the on-nuclear patch-clamp technique did not reveal a regulatory effect of Bok [7], indicating that to see an effect of Bok on IP_3_R1 channel activity requires unperturbed intact cells, where the full complement of feedback regulatory systems are available.

Why have previous studies [7, 14, 16] not shown suppressive effects of Bok on IP_3_R-mediated increases in [Ca^2+^]_C_? Most likely, this is because often the cell types used to measure IP_3_R activity (e.g., MEF and HeLa cells) express IP_3_R3 [8, 70] which cannot bind [11] or be regulated by Bok. In contrast, in the present study, where Bok was shown to have an effect, we used well-defined cell systems in which Ca^2+^ mobilizing activity comes from exogenous or endogenous IP_3_R1 (HEK-3KO and HEK-IP_3_R1 or αT3 and SH-SY5Y, respectively), which binds Bok strongly [20]. Presumably, Bok would have similar effects in cell types (e.g., AR42J cells and hepatocytes [71]) that predominately express IP_3_R2, to which Bok also binds strongly [11].

One study using SV-40 immortalized mouse embryonic fibroblasts (MEFs) from BKO animals showed that BKO disrupts ER-mitochondria contact sites, such that GPCR (ATP and histamine)-induced Ca^2+^ transfer to mitochondria is reduced, but with inconsistent effects on [Ca^2+^]_C_ (only ATP responses were reduced) [14, 15]. A reduction in endogenous IP_3_R levels accompanied BKO, making it hard to ascertain whether the mitochondrial Ca^2+^ transfer deficit was directly due to the loss of Bok, or indirectly due to IP_3_R loss [4, 14]. In contrast, CRISPR-Cas9-mediated BKO in cultured MEFs did not alter endogenous IP_3_R levels or mitochondrial Ca^2+^ transfer [7] suggesting that the Ca^2+^ transfer deficit seen in SV40-immortalized MEFs [14] may have more to do with the loss of IP_3_Rs than Bok. Indeed, in the current study we can attribute the effect of Bok on the post-maximal decline in [Ca^2+^]_C_ to a direct action of Bok on IP_3_R receptor channel activity, since neither BKO nor Bok stable over-expression altered IP_3_R levels, and only those IP_3_Rs that bind Bok were modulated.

It has been noted previously that Bok is phosphorylated at Ser-8 [32, 33], but the present study is the first to characterize the event. Notably, we show that PKA activation through GPCR signaling phosphorylates Bok at Ser-8 in a variety of cell types and that this also weakens its ability to interact with and regulate IP_3_R1. Interestingly, phosphorylation of Mcl-1 weakens its ability to interact with USP9X [72] and phosphorylation of Bcl-2 weakens its ability to interact with Beclin-1 [73], suggesting that weakening of protein-protein interactions is a common effect of Bcl-2 family protein phosphorylation. Ser-8 Bok phosphorylation (or Bok^S8E^) increased Bok immunoreactivity with antibodies where the epitope region is close to position-8, suggesting that Ser-8 phosphorylation leads to a conformational change in the N-terminal region of Bok. This may explain why Ser-8 phosphorylation weakens the ability of Bok to interact with IP_3_R1. Finally, it is tempting to speculate that some of the antibodies raised against phospho-peptides from other proteins might actually recognize epitopes adjacent to the phospho-site, rather than the phosphorylated amino acid itself.

Overall, our results show that Bok suppresses GPCR-induced, IP_3_R1-mediated ER Ca^2+^ release and enhances the post-maximal decline in [Ca^2+^]_C_, which remarkably, is reversed by Bok phosphorylation at Ser-8. Future structural studies on the Bok-IP_3_R1 complex may reveal how Bok inhibits the conformational changes that lead to IP_3_R1 channel opening and ultimately affect IP_3_R1 P_o_. Since IP_3_R1 is by far the predominant IP_3_R subtype in the brain [74], the effects of Bok on IP_3_R-mediated Ca^2+^ mobilization in neuronal cells should be significant [13, 75]. Interestingly, recent studies have shown that Bok is down-regulated in the hippocampus of mouse and human Alzheimer’s disease brains [76, 77] and Bcl-2 family proteins are neuroprotective by suppressing excessive Ca^2+^ signals [78, 79]. It will now be interesting to determine whether the effects of Bok on IP_3_R activity and Ca^2+^ mobilization might contribute to neurodegenerative disorders.

## Electronic supplementary material

Below is the link to the electronic supplementary material.

Supplementary Material 1

Supplementary Material 2

Supplementary Material 3

## Data Availability

No datasets were generated or analysed during the current study.

## Abbreviations

[Ca^2+^]_C_: Cytosolic calcium concentration
[Ca^2+^]_ER_: Endoplasmic reticulum calcium concentration
3KO: IP_3_R1-3 knock-out
ATF-1: Activating Transcription Factor 1
AUC: Area under the curve
Bax: Bcl-2-associated protein x
Bcl-2: B-cell lymphoma 2
Bcl2L10: Bcl-2-like protein 10
Bcl-xL: B-cell lymphoma-extra large
Beclin-1: Bcl-2 interacting protein
BKO: Bok knock-out
Bok: Bcl-2-related ovarian killer
Bok^A^: Anti-Bok^A^
Bok^B^: Anti-Bok^B^
Bok^C^: Anti-Bok^C^
CalA: CalyculinA
CCh: Carbamylcholine, or carbachol
CREB: cAMP-response element binding protein
ER: Endoplasmic reticulum
Fsk: Forskolin
GnRH: Gonadotropin-releasing hormone
GPCR: G protein-coupled receptor
HS-Bok: His-SUMO Bok
IP: Immunoprecipitation
IP_3_: Inositol 1,4,5-triphosphate
IP_3_R: Inositol 1,4,5-triphosphate receptor
Mcl-1: Myeloid cell leukemia-1
MOMP: Mitochondrial outer membrane permeabilization
MS: Mass spectrometry
PACAP: Pituitary adenylate cyclase-activating polypeptide
PGE1: Prostaglandin E1
PKA: Protein kinase A, or cAMP-dependent protein kinase
PP1/2A: Protein phosphatase 1 and 2 A
RRXpS: Anti-PKA substrate
Ser-8: Serine-8
Tg: Thapsigargin
USP9X: Ubiquitin-specific protease 9x
WT: Wild-type
