# Supplementary material for: Phosphorylation of Bok at Ser-8 blocks its ability to suppress IP3R-mediated calcium mobilization
Source: Cell Commun Signal. 2025 Jan 14;23:27. doi: 10.1186/s12964-024-02008-8 (PMC11730779; doi:10.1186/s12964-024-02008-8)
Supplement: Supplementary file 3 — Supplementary Material 3 [file 12964_2024_2008_MOESM3_ESM.docx]

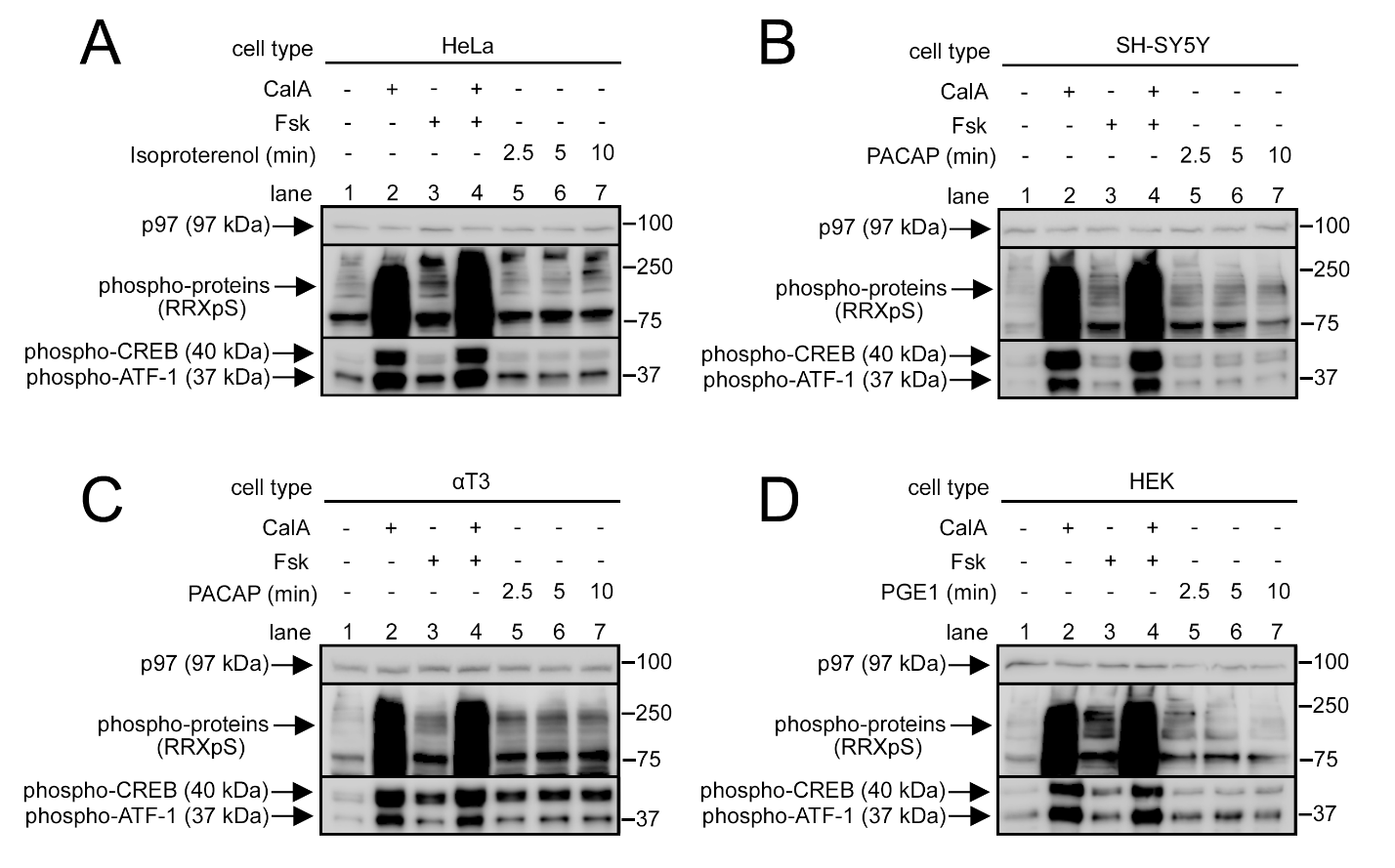


**Supplemental Figure 1) CalA, Fsk, and GPCR agonists increase phospho-CREB and generic phospho-protein levels.** HeLa **(A)**, SH-SY5Y **(B)**, αT3 **(C)**, and HEK **(D)** cells were treated with 100 nM CalA and/or 20 µM Fsk for 10 min, and HeLa cells were treated with 10 µM isoproterenol, SH-SY5Y and αT3 cells were treated with 100 nM PACAP, and HEK cells were treated with 1 µM PGE1, for 2.5, 5, and 10 min. Lysates were probed in immunoblots as indicated, with p97 serving as a loading control. Anti-phospho-CREB recognizes both phospho-CREB and phospho-ATF-1. Compared to control (lane 1), immunoreactivity of phospho-CREB/phospho-ATF-1, as well as phospho-proteins detected with the PKA substrate antibody (RRXpS), increased in all lanes, indicating that all GPCR agonists and drugs increased the levels PKA substrates.


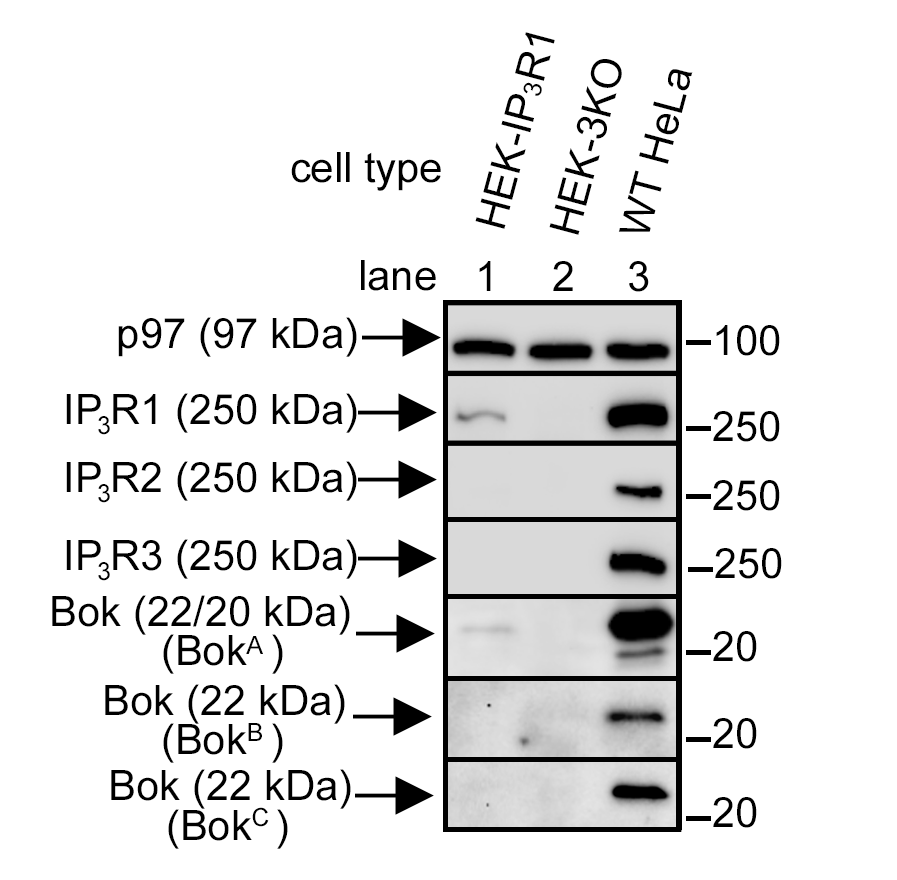


**Supplemental Figure 2) Bok levels in various human cell types.** Lysates were probed in immunoblots as indicated, with p97 serving as a loading control. Endogenous Bok levels in HEK-IP_3_R1 cells that were used in Figures 6-8 (lane 1) and HEK-3KO cells that were used in Figures 3-5 and 8 (lane 2) are very low in comparison to WT HeLa cells that express relatively high levels of endogenous Bok (lane 3). *Note*: there is less endogenous Bok in HEK-3KO cells than HEK-IP_3_R1 cells because Bok stability is dependent on the presence of IP_3_R1 (1).

**
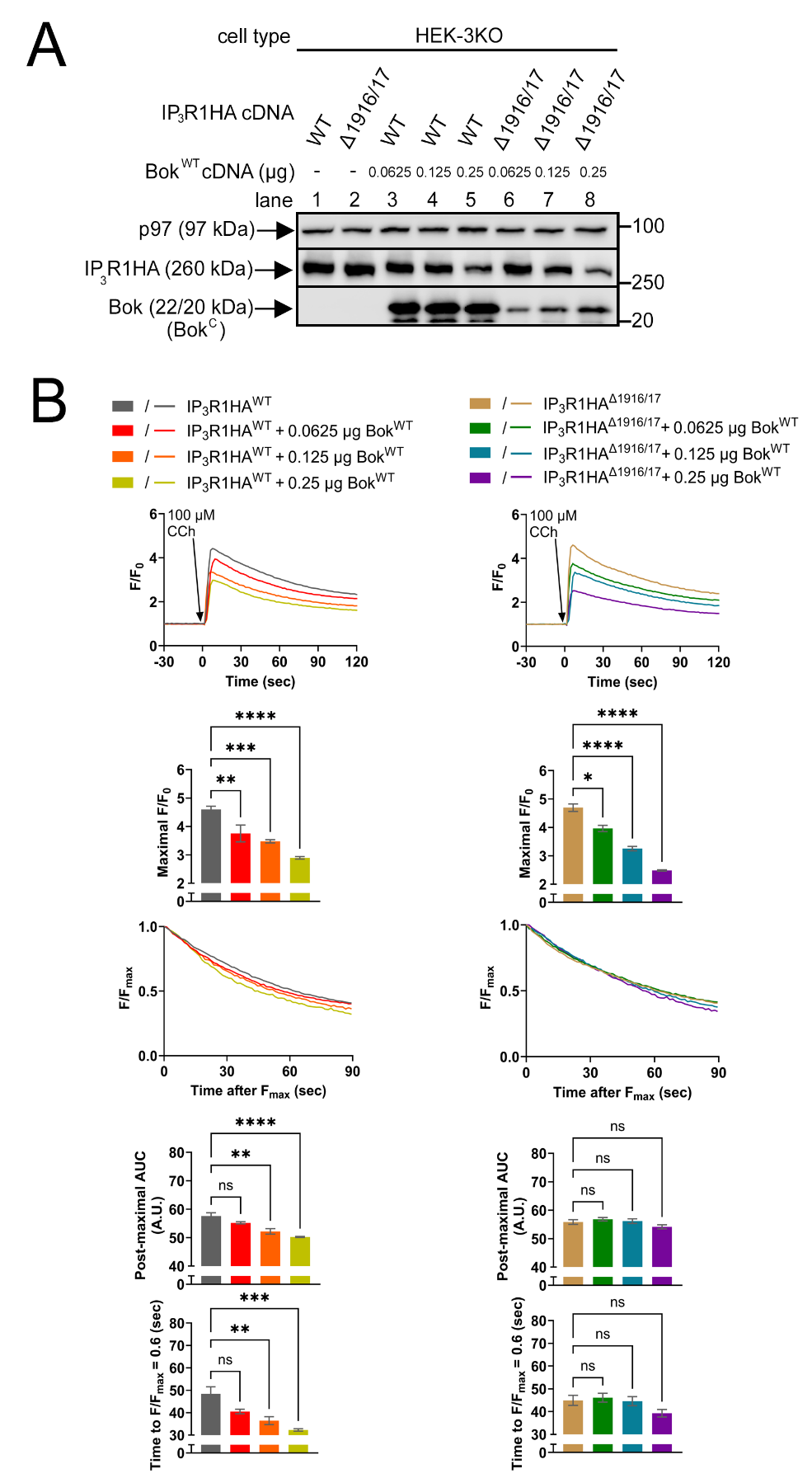
**

**Supplemental Figure 3) Effects of co-transfected Bok on IP_3_R1 expression and [Ca^2+^]_C_ responses in HEK-3KO cells. A),** HEK-3KO cells were transfected with 2 µg IP_3_R1HA^WT^ or 2 µg IP_3_R1HA^Δ1916/17^ with or without increasing amounts of Bok^WT^. Lysates were probed in immunoblots as indicated, with p97 serving as a loading control. Increasing the amount of Bok^WT^ cDNA transfected caused a corresponding decline in both IP_3_R1HA^WT^ and IP_3_R1HA^Δ1916/17^ protein expression. This is most likely due to competition between the mRNAs for translational machinery, rather than an effect of the expressed Bok, since the same decrease is seen for IP_3_R1HA^WT^ and IP_3_R1HA^Δ1916/17^, which cannot bind Bok (2). As expected, Bok^WT^ levels were higher when co-expressed with IP_3_R1HA^WT^ than IP_3_R1HA^Δ1916/17^ (lanes 3-5 vs 6-8), because Bok stability is dependent on binding to IP_3_R1 (1). **B),** [Ca^2+^]_C_ (F/F_0_) in HEK-3KO cells transfected with IP_3_R1HA^WT^ (left panels) or IP_3_R1HA^Δ1916/17^ (right panels) with or without increasing amounts of Bok^WT^, exposed to 100 µM CCh, added at t=0. The maximal F/F_0_ values (F_max_), post-maximal decline in [Ca^2+^]_C_, post-maximal area under the curve (AUC), and time to F/F_max_ = 0.6 are shown underneath the corresponding IP_3_R1 construct (mean ± SEM, n=3, *, **, ***, and **** designates p<0.05, p<0.005, p<0.0005, and p<0.00005, respectively, ns = not significant, p>0.05). These data show that increasing the amount of Bok^WT^ cDNA transfected caused a corresponding decrease in the F_max_ values with both IP_3_R1 constructs, but that only IP_3_R1HA^WT^ responds with a significantly altered post-maximal decline in [Ca^2+^]_C_.

**
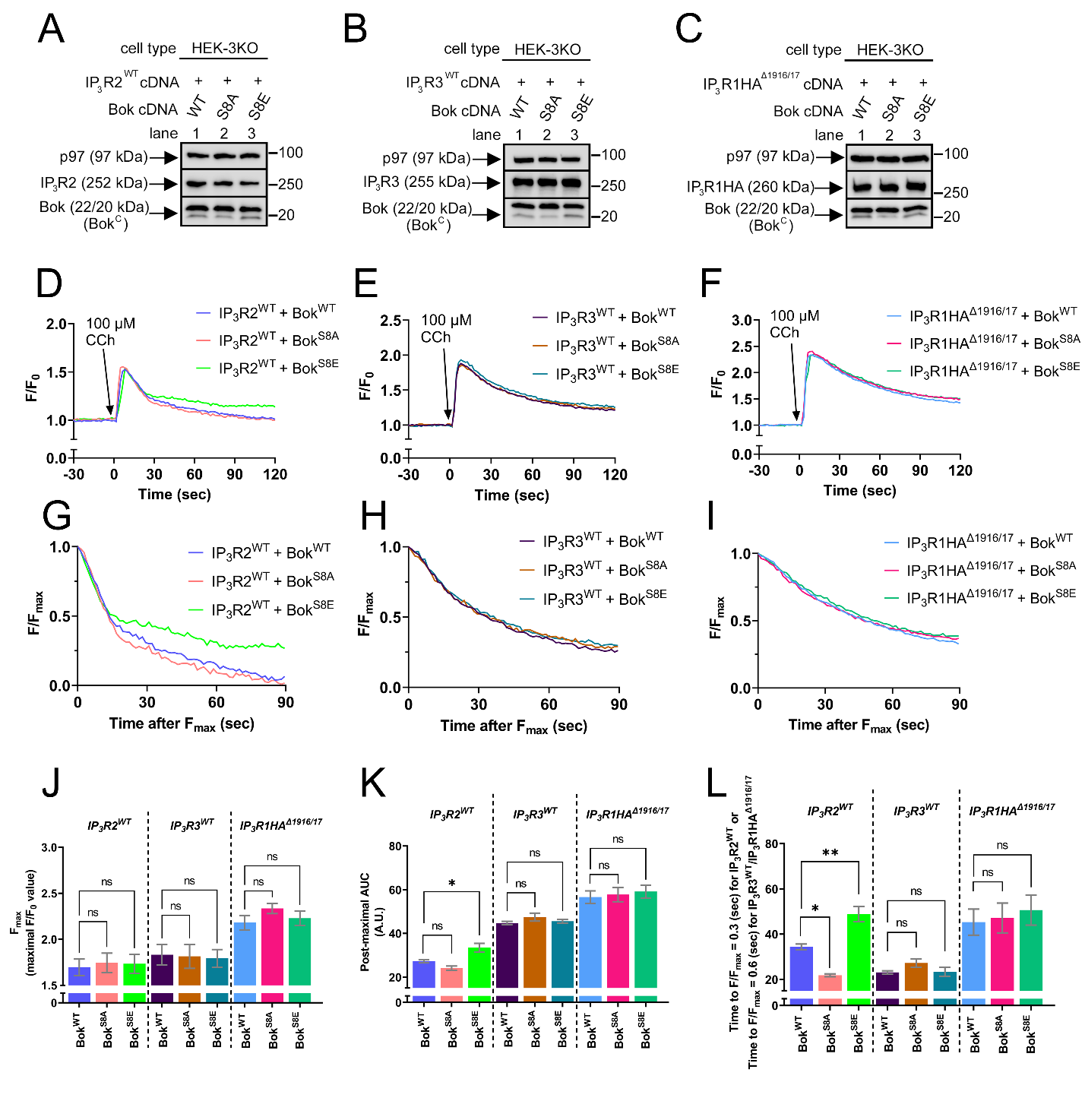
Supplemental Figure 4) Differential effects of Bok^WT^, Bok^S8A^, and Bok^S8E^ on the post-maximal decline in [Ca^2+^]_C_ are seen only with IP_3_Rs that can bind Bok (i.e., IP_3_R2^WT^, but not IP_3_R3^WT^ and IP_3_R1HA^Δ1916/17^). A-C),** HEK-3KO cells were transfected to express IP_3_R2^WT^, IP_3_R3^WT^, or IP_3_R1HA^Δ1916/17^, and either Bok^WT^, Bok^S8A^, or Bok^S8E^. Lysates were probed in immunoblots as indicated, with p97 serving as a loading control. **D-E),** [Ca^2+^]_C_ (F/F_0_) in transfected HEK-3KO cells exposed to 100 µM CCh, added at t=0. **G-I),** Post-maximal decline in [Ca^2+^]_C_ graphed as F/F_max_. **J-L),** Maximal F/F_0_ values (F_max_), post-maximal area under the curve (AUC), and time to F/F_max_ = 0.3 for IP_3_R2^WT^ or time to F/F_max_ = 0.6 for IP_3_R3^WT^ and IP_3_R1HA^Δ1916/17^ (mean ± SEM, n=3, * and ** designates p<0.05 and p<0.005, respectively, ns = not significant, p>0.05).


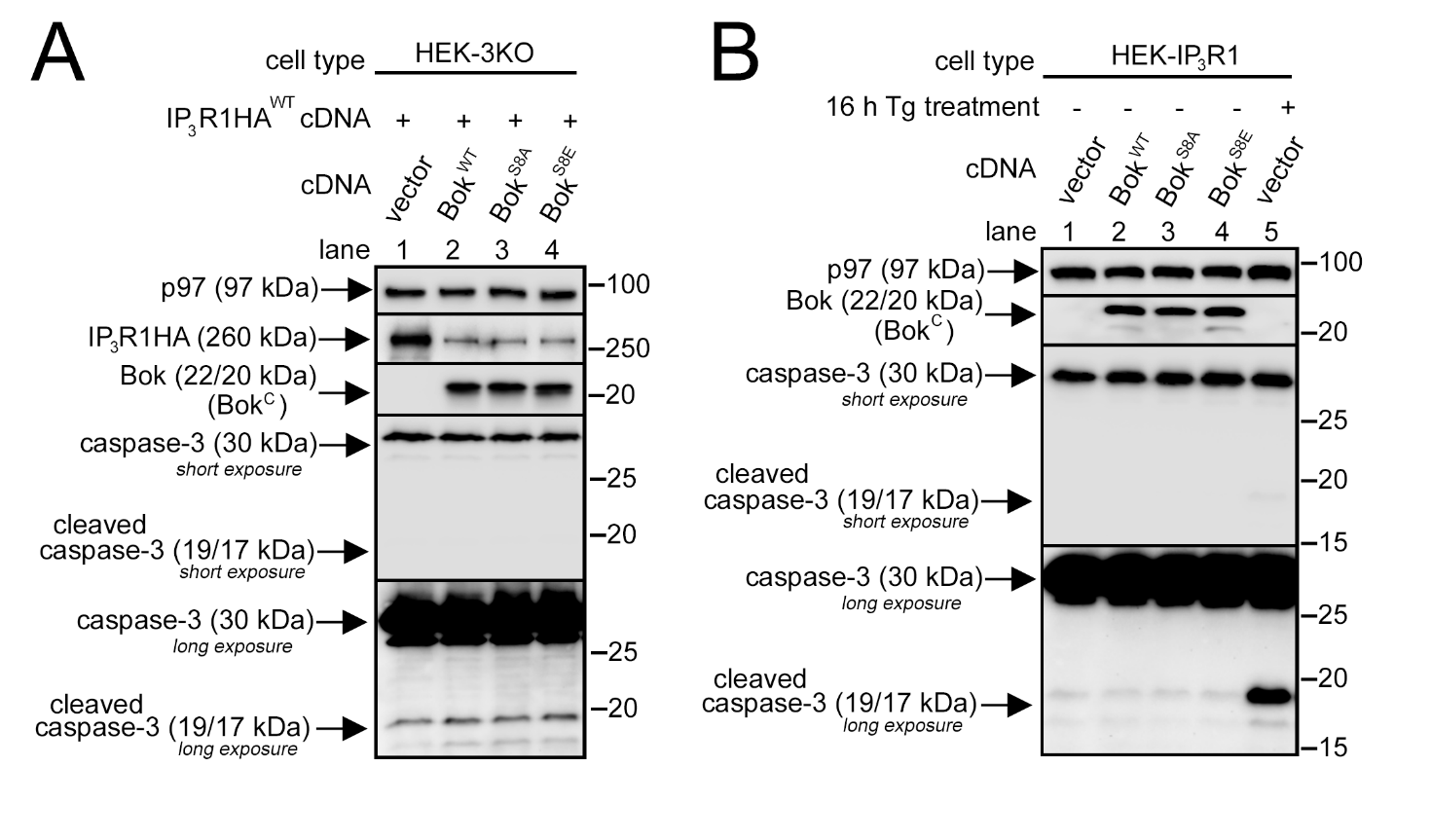


**Supplemental Figure 5) Exogenous Bok expression in HEK cells has little or no effect on apoptotic signaling. A),** HEK-3KO cells were transfected as in Figure 5 to express IP_3_R1HA^WT^, and either Bok^WT^, Bok^S8A^, or Bok^S8E^, with vector as a control. Lysates were probed in immunoblots as indicated, with p97 serving as a loading control. There is some Bok-induced apoptosis, as indicated by a slight elevation in cleaved caspase-3 (lanes 2-4 vs 1), however, this cleavage is equal for Bok^WT^, Bok^S8A^, and Bok^S8E^, indicating that apoptosis is not a factor in the differential [Ca^2+^]_C_ responses seen in Figure 5. **B),** Lysates from HEK-IP_3_R1 cells stably expressing either Bok^WT^, Bok^S8A^, or Bok^S8E^, with vector as a control, as in Figure 6, were probed in immunoblots as indicated, with p97 serving as a loading control. Vector-transfected cells exposed to 1 µM Tg for 16 h (lane 5) served as a positive control for caspase-3 cleavage. There was no Bok-induced caspase-3 cleavage (lanes 1-4), indicating that apoptosis is not a factor in the [Ca^2+^]_C_ or [Ca^2+^]_ER_ changes seen in Figures 6 and 7, respectively.

**
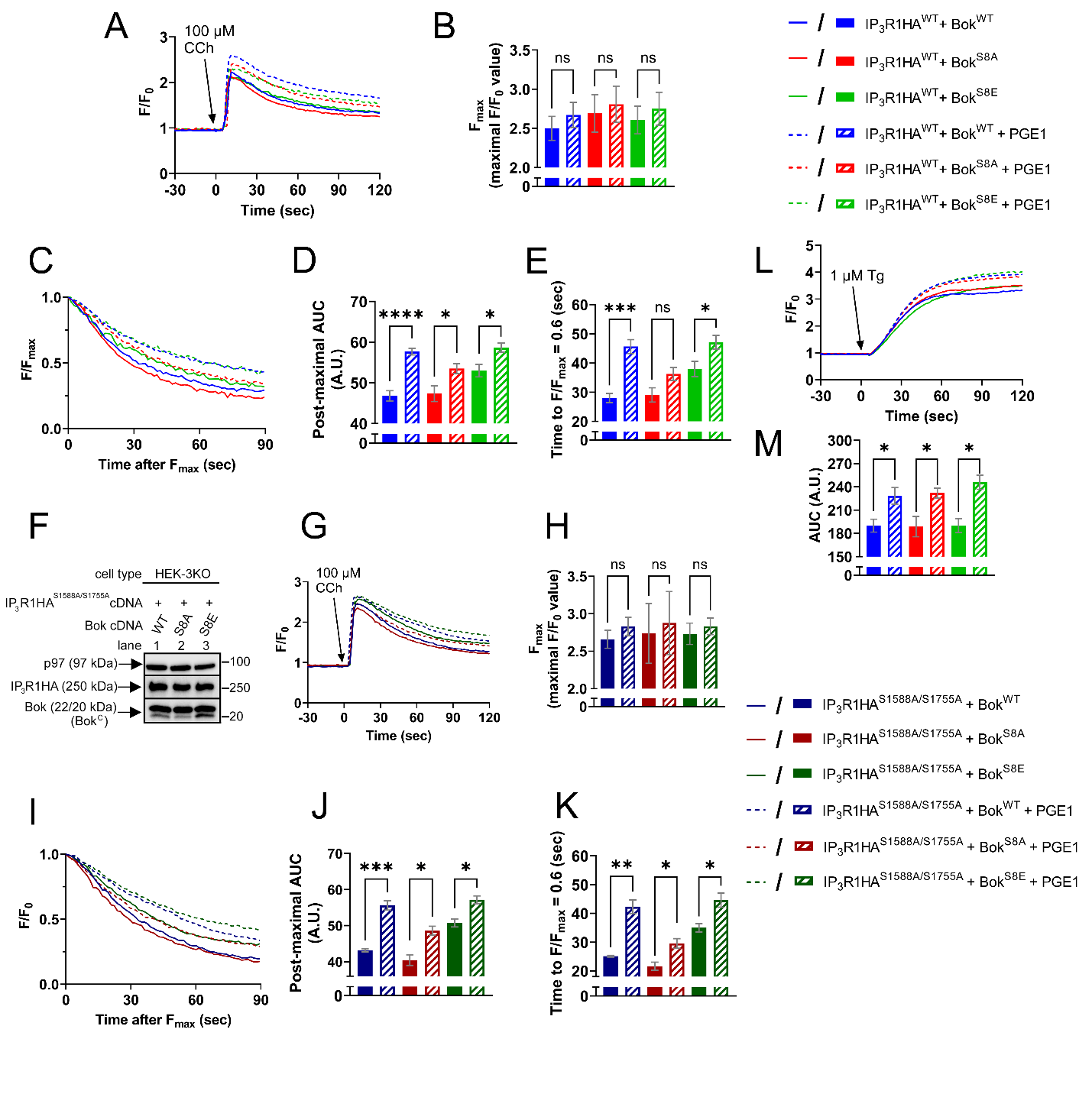
Supplemental Figure 6) PGE1 enhancement of IP_3_R1-mediated Ca^2+^ mobilization is due to an increase in ER Ca^2+^ store size and not Bok phosphorylation at Ser-8 or IP_3_R1 phosphorylation at Ser-1588 and Ser-1755. A),** [Ca^2+^]_C_ (F/F_0_) in HEK-3KO cells co-expressing IP_3_R1HA^WT^ and either Bok^WT^, Bok^S8A^, or Bok^S8E^, pre-treated without (solid lines) or with (dashed lines) 1 µM PGE1 for 2.5 min prior to 100 µM CCh, added at t=0. PGE1 enhanced [Ca^2+^]_C_ under all conditions. **B),** Maximal F/F_0_ values (F_max_) in non-treated (solid bars) or PGE1-treated cells (striped bars), compiled from data shown in Figure 5C and H, showing that PGE1 slightly, but non-significantly, increases F_max_ regardless of Bok construct (mean ± SEM, n=5, ns = not significant). **C),** Post-maximal decline in [Ca^2+^]_C_ graphed as F/F_max_ in non-treated or PGE1-treated cells. **D) and E),** Post-maximal area under the curve (AUC) and time to F/F_max_ = 0.6 in non-treated or PGE1-treated cells (mean ± SEM, n=5, *, ***, and **** designates p<0.05, p<0.0005, and p<0.00005, respectively, ns = not significant, p>0.05), compiled from data shown in Figure 5E and J for post-maximal AUC and Figure 5F and K for time to F/F_max_ = 0.6. Together, panels A-E show that PGE1 had a general enhancing effect on the CCh-induced increases in [Ca^2+^]_C_, in a manner that is independent of Bok phosphorylation at Ser-8. **F),** HEK-3KO cells were transfected to express IP_3_R1HA^S1588A/S1755A^ and either Bok^WT^, Bok^S8A^, or Bok^S8E^. Lysates were probed in immunoblots as indicated, with p97 serving as a loading control. **G-K),** Parallel analysis of [Ca^2+^]_C_ responses in cells expressing IP_3_R1HA^S1588A/S1755A^ and either Bok^WT^, Bok^S8A^, or Bok^S8E^ (mean ± SEM, n=3, *, **, and *** designates p<0.05, p<0.005, and p<0.0005, respectively, ns = not significant). Together, panels G-K show that PGE1 had a general enhancing effect on the CCh-induced increases in [Ca^2+^]_C_, in a manner that is independent of IP_3_R1 phosphorylation at Ser-1588 and Ser-1788, since IP_3_R1HA^S1588A/S1755A^ was affected by PGE1 just like IP_3_R1HA^WT^. **L),** [Ca^2+^]_C_ (F/F_0_) in HEK-3KO cells transfected to express IP_3_R1HA^WT^ and either Bok^WT^, Bok^S8A^, or Bok^S8E^, pre-treated without (solid lines) or with (dashed lines) 1 µM PGE1 for 2.5 min prior to 1 µM Tg added at t=0. **M),** Area under the curve (AUC) in non-treated or PGE1-treated cells (mean ± SEM, n=3, * designates p<0.05). In all cells, PGE1 enhances Tg-induced [Ca^2+^]_C_ indicating that PGE1 increases the ER Ca^2+^ store size, which is not dependent on Bok phosphorylation at Ser-8. This result likely explains why PGE1 enhances [Ca^2+^]_C_ after CCh exposure.


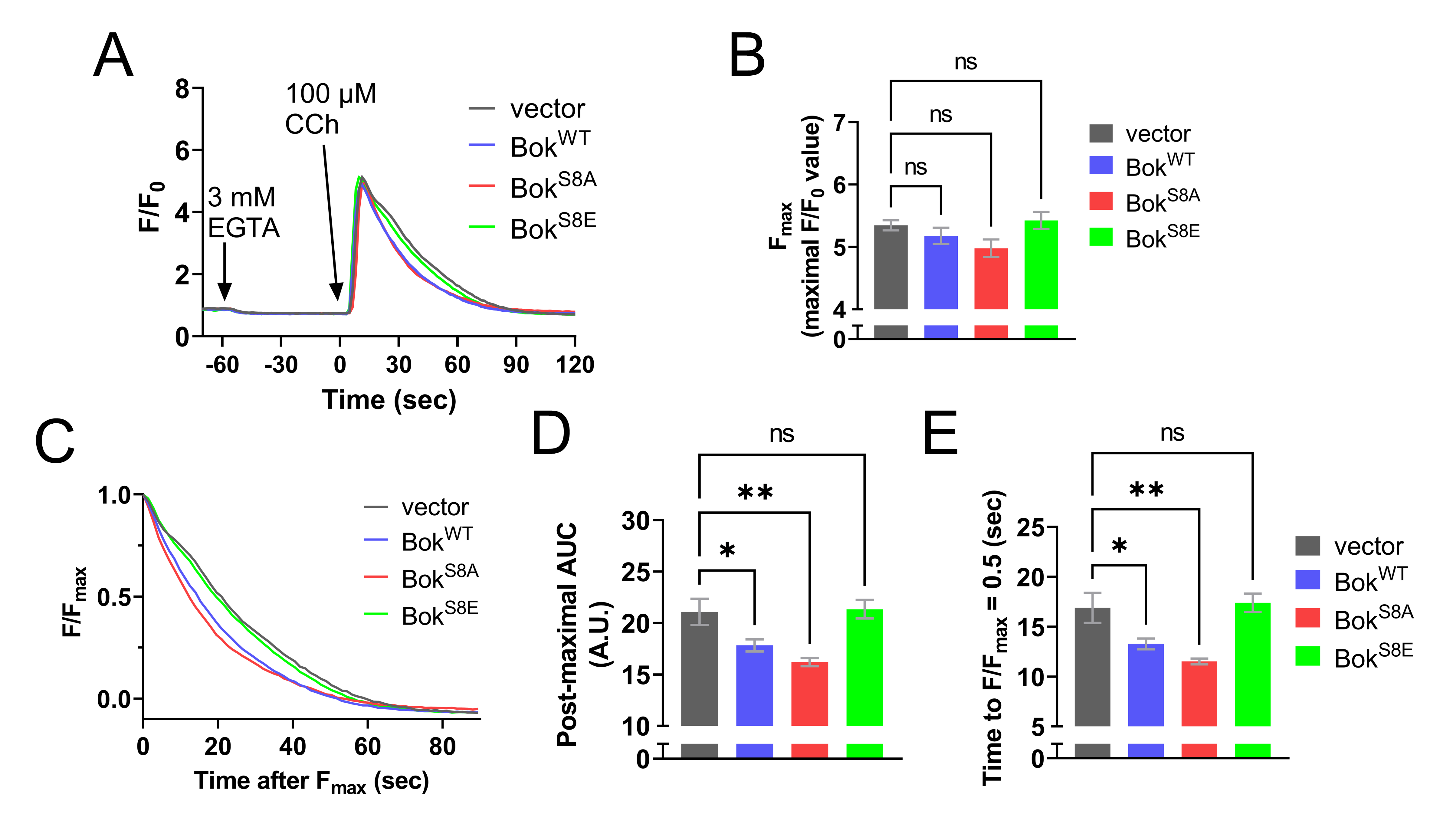


**Supplemental Figure 7) Elimination of Ca^2+^ entry does not change the ability of Bok to accelerate the post-maximal decline in [Ca^2+^]_C_*.*** **A),** [Ca^2+^]_C_ (F/F_0_) in HEK-IP_3_R1 cells stably expressing either Bok^WT^, Bok^S8A^, or Bok^S8E^, with vector as a control, exposed to 3 mM EGTA, added 60 sec prior to the addition of 100 µM CCh, added at t=0. 3 mM EGTA reduces extracellular [Ca^2+^] to ~100 nM and reduced [Ca^2+^]_C_ by ~20%, indicating Ca^2+^ entry was blocked (3). **B),** Maximal F/F_0_ values (F_max_) (mean ± SEM, n=4, ns = not significant, p>0.05). **C),** Post-maximal decline in [Ca^2+^]_C_ graphed as F/F_max_. **D) and E),** Post-maximal area under the curve (AUC) and time to F/F_max_ = 0.5 (mean ± SEM, n=4, * and ** designates p<0.05 and p<0.005, respectively, ns = not significant, p>0.05). **F-J),** Parallel analysis of cells pretreated with 1 µM PGE1 for 1.5 min prior to EGTA addition and 2.5 min prior to CCh addition (mean ± SEM, n=4, *** designates p<0.0005, respectively, ns = not significant, p>0.05).


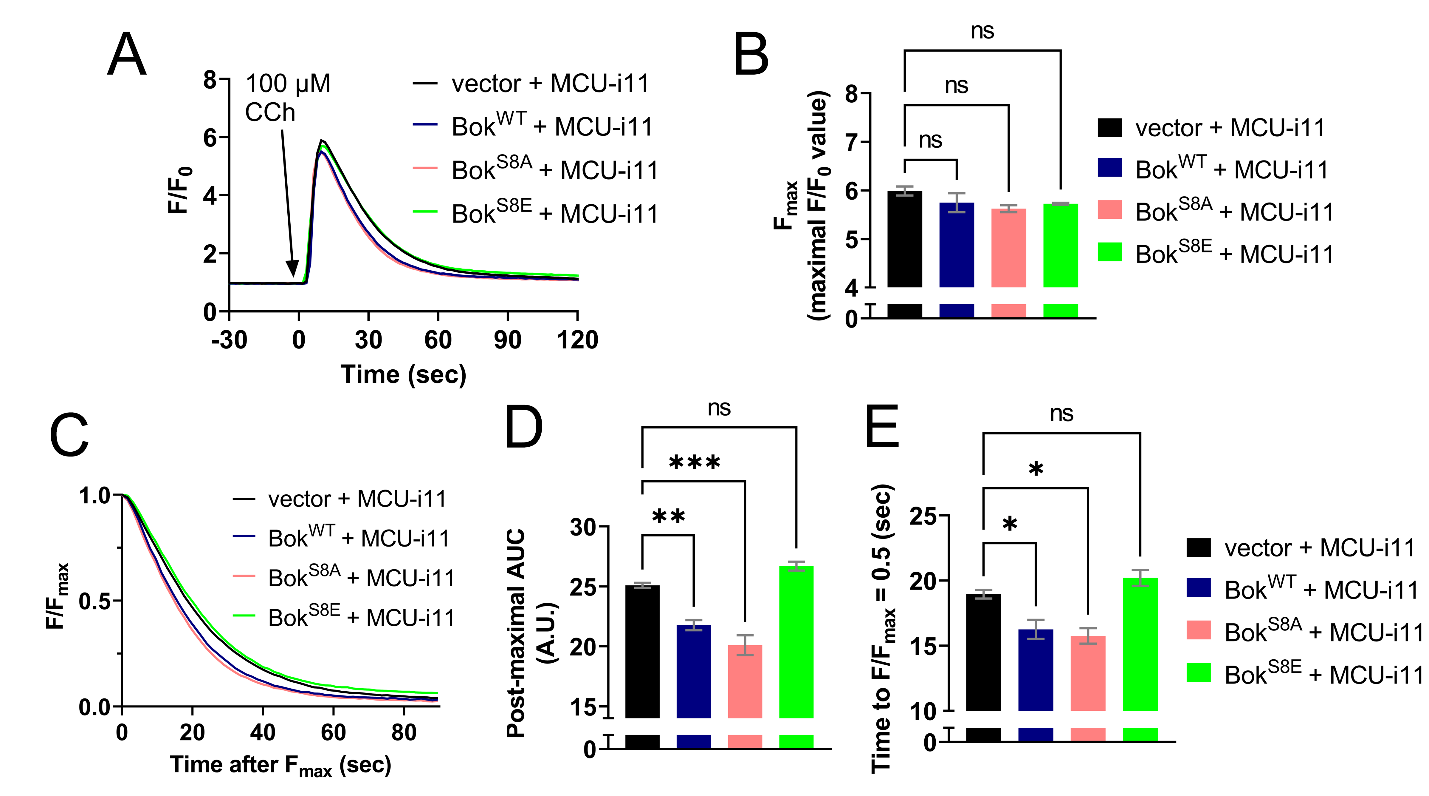


**Supplemental Figure 8) Inhibition of mitochondrial Ca^2+^ uptake with MCU-i11 does not change the ability of Bok to accelerate the post-maximal decline in [Ca^2+^]_C_*.*** **A),** [Ca^2+^]_C_ (F/F_0_) in HEK-IP_3_R1 cells stably expressing either Bok^WT^, Bok^S8A^, or Bok^S8E^, with vector as a control, exposed to 10 µM MCU-i11, added 120 sec prior to the addition of 100 µM CCh, added at t=0. 10 µM MCU-i11 significantly inhibits mitochondrial Ca^2+^ uptake in HEK cells (4, 5). **B),** Maximal F/F_0_ values (F_max_) (mean ± SEM, n=3, ns = not significant, p>0.05). **C),** Post-maximal decline in [Ca^2+^]_C_ graphed as F/F_max_. **D) and E),** Post-maximal area under the curve (AUC) and time to F/F_max_ = 0.5 (mean ± SEM, n=3, *, **, *** designates p<0.05, <0.005, and <0.0005, respectively, ns = not significant, p>0.05).


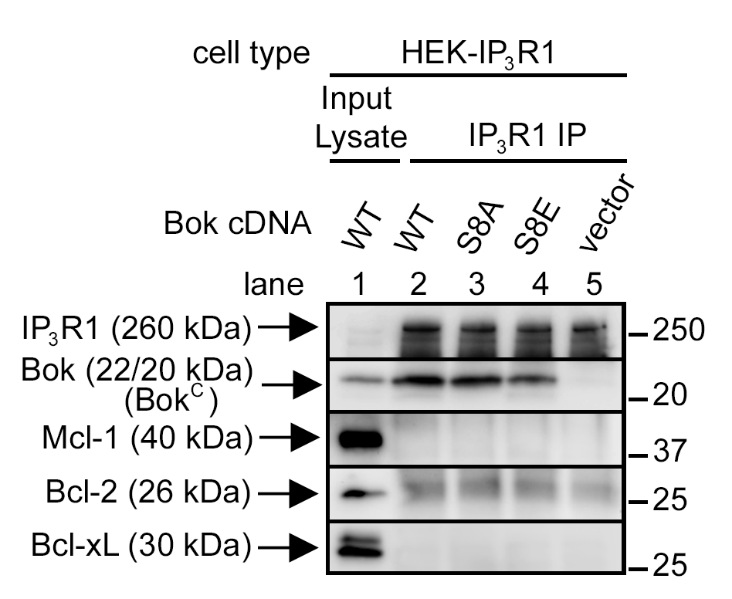


**Supplemental Figure 9) Lack of effect of Bok construct expression on potential interactions with Bcl-2 family members and the Bok-IP_3_R1 complex.** In parallel to Figure 8B, IP_3_R1 IPs from HEK-IP_3_R1 cells stably expressing Bok^WT^, Bok^S8A^, or Bok^S8E^, with vector as a control, were probed in immunoblots for various Bcl-2 family members (lanes 2-5), with lysate from HEK-IP_3_R1 cells stably expressing Bok^WT^ serving as a control to show the relative expression level of proteins in HEK-IP_3_R1 cells (lane 1). Mcl-1, Bcl-2, and Bcl-xL were examined since many studies have shown that these proteins regulate IP_3_R1 activity (6), although none bind to IP_3_Rs nearly as strongly as Bok (7). In all cell types (lanes 2-5), Mcl-1, Bcl-2, and Bcl-xL fail to co-IP with the Bok-IP_3_R1 complex indicating that the effect of Bok^WT^ and the lack of effect of Bok^S8E^ on [Ca^2+^]_C_ and [Ca^2+^]_ER_ cannot be explained by interactions between other Bcl-2 family members and the Bok-IP_3_R1 complex.

**
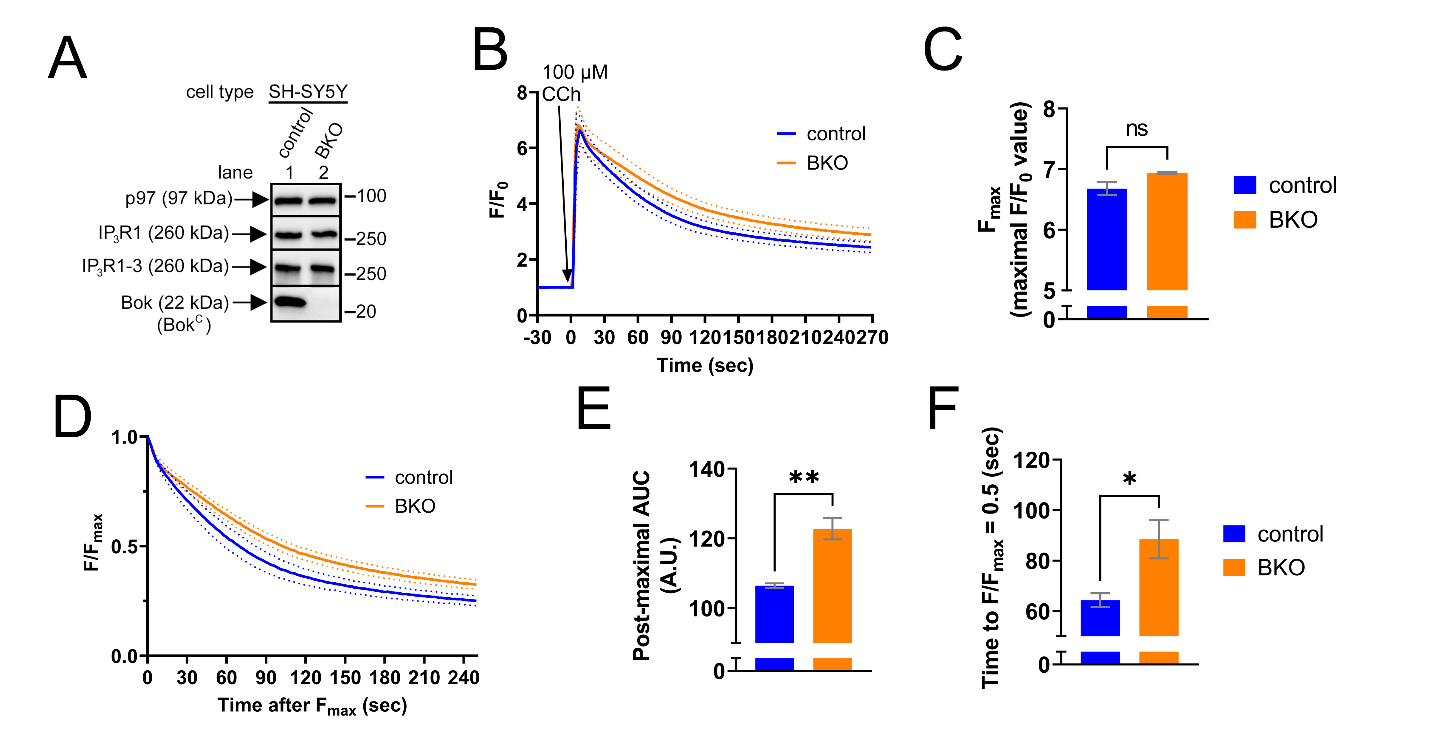
**

**Supplemental Figure 10) Endogenous Bok accelerates the post-maximal decline in** **[Ca^2+^]_C_ in SH-SY5Y cells*.*** **A),** The CRISPR-Cas9 system using the pCas-Guide-EF1a-GFP vector (#GE100018, OriGene) encoding the following gRNAs: AAAGGCGTCCATGATCTCGG, GTCTGTGGGCGAGCGGTCAA, and GCCCCGCGGCCACCGCATAC, was used to generate BKO SH-SY5Y cell lines. Briefly, WT SH-SY5Y cells were transfected using Lipofectamine 2000 and 48 h later GFP-expressing cells were selected by fluorescence-activated cell sorting and plated at 1 cell/well in a 96-well plate. Colonies were expanded and screened in immunoblots for loss of Bok protein as described (8). Colonies in which BKO failed were considered control cells. Representative lysates from one control and one BKO cell line were probed for Bok, as well as IP_3_R1 and IP_3_R1-3 to demonstrate loss of Bok protein did not change IP_3_R1 or total IP_3_R expression, with p97 serving as a loading control. **B),** [Ca^2+^]_C_ (F/F_0_) in SH-SY5Y cells exposed to 100 µM CCh, added at t=0. Traces shown are mean ± SEM from four control and four BKO cell lines from a representative experiment. **C),** Maximal F/F_0_ values (F_max_) (mean ± SEM, n=3, ns = not significant, p>0.05). **D****),** Post-maximal decline in [Ca^2+^]_C_ graphed as F/F_max_. Traces shown are mean ± SEM from four control and four BKO cell lines from the same representative experiment in panel B. **E) and F),** Post-maximal area under the curve (AUC) and time to F/F_max_ = 0.5 (mean ± SEM, n=3, * and ** designates p<0.05 and p<0.005, respectively).

**References:**

1. Bonzerato CG, Keller KR, Schulman JJ, Gao X, Szczesniak LM, Wojcikiewicz RJH. Endogenous Bok is stable at the endoplasmic reticulum membrane and does not mediate proteasome inhibitor-induced apoptosis. Front Cell Dev Biol. 2022;10:1094302.

2. Szczesniak LM, Bonzerato CG, Schulman JJ, Bah A, Wojcikiewicz RJH. Bok binds to a largely disordered loop in the coupling domain of type 1 inositol 1,4,5-trisphosphate receptor. Biochem Biophys Res Commun. 2021;553:180-6.

3. Gao X, Keller KR, Bonzerato CG, Li P, Laemmerhofer M, Wojcikiewicz RJH. The ubiquitin-proteasome pathway inhibitor TAK-243 has major effects on calcium handling in mammalian cells. Biochim Biophys Acta Mol Cell Res. 2024;1871(1):119618.

4. Di Marco G, Vallese F, Jourde B, Bergsdorf C, Sturlese M, De Mario A, et al. A High-Throughput Screening Identifies MICU1 Targeting Compounds. Cell Rep. 2020;30(7):2321-31 e6.

5. Marta K, Hasan P, Rodriguez-Prados M, Paillard M, Hajnoczky G. Pharmacological inhibition of the mitochondrial Ca(2+) uniporter: Relevance for pathophysiology and human therapy. J Mol Cell Cardiol. 2021;151:135-44.

6. Ivanova H, Vervliet T, Monaco G, Terry LE, Rosa N, Baker MR, et al. Bcl-2-Protein Family as Modulators of IP3 Receptors and Other Organellar Ca(2+) Channels. Cold Spring Harb Perspect Biol. 2020;12(4).

7. Bonzerato CG, Wojcikiewicz RJH. Bok: real killer or bystander with non-apoptotic roles? Front Cell Dev Biol. 2023;11:1161910.

8. Schulman JJ, Szczesniak LM, Bunker EN, Nelson HA, Roe MW, Wagner LE, 2nd, et al. Bok regulates mitochondrial fusion and morphology. Cell Death Differ. 2019;26(12):2682-94.
